# Application of Carbon-Based Catalysts Derived from Ship Antifouling Paint Particles in Ultrasound-Fe^2+^/Peroxydisulfate Advanced Oxidation Process for Activated Sludge Reduction: A Pilot-Scale Study

**DOI:** 10.3390/toxics14040292

**Published:** 2026-03-28

**Authors:** Can Zhang, Kunkun Yu, Jianhua Zhou, Deli Wu

**Affiliations:** 1 Department of Environmental Science and Engineering, Tongji University, No. 1239 Siping Road, Shanghai 200092, China; 2280242@tongji.edu.cn; 2 COSCO Shipping Heavy Industry Co., Ltd., No. 628 Minsheng Road, Shanghai 200135, China; 3 COSCO Shipping Heavy Industry (Weihai) Technology Co., Ltd., No. 9 South Shenyang Road, Weihai 264200, China

**Keywords:** ship antifouling paint particles, resource utilization, carbon-based catalyst, Fe^2+^/peroxydisulfate, activated sludge reduction

## Abstract

Activated sludge treatment is plagued by high secondary pollution risks, and ship antifouling paint particles (APPs) as hazardous heavy metal-rich solid wastes generated from hull derusting wastewater, pose severe environmental threats and intractable disposal dilemmas. This study developed a novel pilot-scale activated sludge reduction process coupling APPs-derived carbon-based catalysts with ultrasound-Fe^2+^/peroxydisulfate (PDS) advanced oxidation. Columnar catalysts were fabricated via direct carbonization-molding using waste APPs from an 82,000 deadweight bulk carrier were used as the sole raw material to prepare columnar catalysts via direct carbonization-molding; single-factor and orthogonal experiments optimized process parameters, Scanning Electron Microscopy (SEM), Energy Dispersive Spectroscopy (EDS) and X-ray Photoelectron Spectroscopy (XPS) characterized catalyst and sludge properties, free radical quenching experiments elucidated reaction mechanisms and a 90-day continuous pilot run assessed catalytic stability. The process achieved a 43.5% sludge removal rate under optimal conditions, accompanied by 100% toluene and 92.3% phenolic compound degradation, as well as efficient total phosphorus (TP) and total nitrogen (TN) removal. Mechanistic studies via characterization and quenching experiments confirmed the catalyst enhanced PDS activation through free/non-free radical synergy and accelerated Fe^2+^/Fe^3+^ redox cycling. A 90-day continuous pilot operation demonstrated excellent long-term catalytic stability, with sludge removal rate remaining above 38%. This “waste treating waste” technology realizes high-value APPs resource utilization, provides a low-carbon sludge disposal pathway, and offers a scalable solution for collaborative pollution control in the wastewater treatment and shipping industries.

## 1. Introduction

Activated sludge, a major by-product of municipal wastewater treatment composed of microorganisms, extracellular polymeric substances (EPS), bound water and various pollutants, has a dense EPS barrier that exacerbates hydration and hinders dewatering and reduction [1,2]. With urbanization and upgraded wastewater treatment standards, sludge output is surging, and its enrichment of pathogenic bacteria, heavy metals and refractory carcinogens poses severe secondary pollution risks due to low harmless disposal rates [3]. Mainstream disposal methods (incineration, landfilling) suffer from leachate pollution, high carbon emissions and policy restrictions, leading to mounting disposal pressure [4]. Thus, developing an efficient, economical and eco-friendly sludge reduction technology is critical for advancing green and low-carbon development of the wastewater treatment industry.

Physical and chemical conditioning are mainstream sludge reduction technologies, yet single applications have inherent limitations. Ultrasonic pretreatment, a promising physical method, utilizes cavitation-induced local high temperature and shear force to destroy sludge floc structure, release bound water and intracellular organics for preliminary reduction, with the advantages of simple operation and no secondary pollution [5]. The Fe^2+^/PDS Fenton-like advanced oxidation technology outperforms traditional Fenton processes in reaction rate, pH adaptability and oxidation capacity, generating highly oxidizing sulfate radicals (SO_4_·^−^, E^0^ = 2.5–3.1 V) and hydroxyl radicals (·OH, E^0^ = 2.8 V) to degrade sludge EPS and organics [6,7]. However, the single Fe^2+^/PDS system is limited in engineering application by low PDS utilization, difficult Fe^2+^ recovery and incomplete organic mineralization [8]. Studies have confirmed that carbon-based materials loaded with transition metals can synergistically enhance PDS activation via carrier and co-catalytic effects, providing a new idea for preparing high-performance catalysts from solid wastes to overcome the defects of single oxidation systems [9,10].

In the shipping industry, ship-hull coating systems typically consist of three layers: primer, tie coat and topcoat, which together ensure surface durability and service life. The primer enhances adhesion and corrosion resistance, while the tie coat can strengthen the bonding between the primer and the topcoat [11,12]. The topcoat, also known as antifouling paint, is formulated with organometallic fungicides and organic copolymers. Following application, biocides leach gradually into surrounding seawater, preventing the attachment of fouling organisms [13,14]. Antifouling technologies have undergone three generations of antifouling paint upgrades: (1) early soluble antifouling coatings based on rosin or rosin derivative-crosslinked CuO, (2) tin acrylate polymers [15,16,17], which have been mandatorily banned by the International Maritime Organization (IMO) in 2008 due to (IMO 2019), and (3) the currently widely used acrylate copolymers with copper, ferric, zinc, or silane on side chains replacing organotin [18,19,20]. Long-term marine operation causes hull corrosion, necessitating derusting before repainting [21]. Ultra-high pressure water jet derusting, the mainstream technology, generates ship antifouling paint particles (APPs) that are either collected or discharged with derusting wastewater [22,23]. Listed as a potential marine microplastic source by the International Union for Conservation of Nature (IUCN), APPs are regulated as hazardous waste for on-site collection and disposal, yet unified disposal standards and mature technologies are lacking [24]. Actual direct discharge of derusting wastewater causes severe environmental risks: APPs are highly toxic due to Cu/Fe metals and biocides, their non-biodegradable plastic matrix persists in the marine environment for decades, and biocide leaching endangers coastal ecosystems [25,26,27]. Landfilling or random stacking leads to heavy metal leaching and soil/water pollution, making APPs disposal an urgent environmental challenge [28].

Notably, APPs possess natural advantages as carbon-based catalyst raw materials: their intrinsic Cu/Fe metals are high-quality transition metal active components for PDS activation, and their carbonaceous matrix can be converted into carbon-based materials via carbonization with PDS activation and carrier functions. Preparing carbon-based catalysts from APPs abandons reliance on commercial activated carbon and additional metal precursors, realizing high-value resource conversion of hazardous waste. Based on this, this study prepared columnar carbon-based catalysts using waste APPs as the sole raw material via direct carbonization-molding and constructed a novel sludge reduction process coupling APPs-derived catalysts with ultrasound-Fe^2+^/PDS oxidation. Single-factor and orthogonal tests were used to optimize operational parameters; multiple characterization techniques and free radical quenching experiments were employed to analyze sludge and catalyst structural changes and clarify the synergistic reduction mechanism.

This study aims to provide a new technical pathway for efficient municipal sludge reduction, solve the disposal dilemma of waste ship APPs, and realize the dual environmental benefits of waste resource reuse and sludge deep reduction. It also offers a reference for pollution control and resource utilization of similar heavy metal-rich industrial solid wastes and promotes collaborative green development of the wastewater treatment and shipping industries.

## 2. Materials and Methods

### 2.1. Experimental Materials

#### 2.1.1. Raw Activated Sludge

Raw activated sludge was collected from the aeration tank of a municipal wastewater treatment plant in Weihai city, Shandong Province, China, with a daily treatment capacity of 100,000 m^3^. The sludge was grayish-black in appearance and homogenized via continuous stirring for 24 h in a storage tank (effective volume: 5 m^3^) to ensure uniform physicochemical properties before use. Its basic characteristics were determined under room temperature and atmospheric pressure, as presented in Table 1.

#### 2.1.2. Chemical Reagents

Ferrous sulfate (FeSO_4_, Analytical Reagent, ≥99.0%), sodium peroxydisulfate (Na_2_S_2_O_8_, Analytical Reagent, ≥99.0%), tert-butanol (TBA, Analytical Reagent, ≥99.0%) and methanol (MeOH, Analytical Reagent, ≥99.5%) were purchased from Sinopharm Chemical Reagent Co., Ltd. (Shanghai, China). Ultrapure water (18.2 MΩ·cm) used for reagent preparation was produced by a Milli-Q water purification system (Millipore, Bedford, MA, USA).

All chemical reagents were used as received without further purification. Large-volume reagent solutions (FeSO_4_: 1000 L batches; Na_2_S_2_O_8_: 1000 L batches) were prepared in dedicated mixing tanks to meet the continuous operation demand. All glassware were rinsed with ultrapure water three times before use to avoid contamination.

#### 2.1.3. Ship Antifouling Paint Particles (APPs)

Derusting wastewater was sampled during ultra-high-pressure water jet derusting of an 82,000 dead weight tonnage bulk carrier at a ship repair yard’s dock in Nantong, China. The wastewater, mainly containing suspended APPs and iron rust with a solid content of 22 ± 1.7 g·L^−1^, was collected via a closed vacuum recovery system into a dedicated tank. A total of 50 m^3^ of well-mixed wastewater was sampled at the stable derusting stage to obtain representative APPs raw material for catalyst preparation.

APPs were extracted and pretreated from the collected derusting wastewater via solid-liquid separation combined with magnetic separation, with the detailed process provided in the Supplementary Information S1. Briefly, 50 m^3^ derusting wastewater first underwent gravity sedimentation for 3 h, and the concentrated slurry was pressure-filtered through the plate-and-frame filter (0.6 MPa, 5 μm polypropylene filter cloth) to collect the filter cake. The dried cake was processed by a 1.2 T high-intensity magnetic separator (Shandong Luci Magnetic Industry Technology Co., Ltd., Linyi, Shandong, China) to remove iron rust, with the non-magnetic APPs fraction collected at a recovery rate of 82.3%. The obtained APPs were crushed and sieved to collect the <100 μm particle size fraction as the final raw material. Through this process, 814.6 kg of qualified APPs raw material was obtained, satisfying the requirements for catalyst preparation and related characterization tests.

### 2.2. Preparation of APPs-Derived Carbon-Based Catalyst

#### 2.2.1. Molding Aid Optimization and Mixing

Starch was used as a shaping agent, with 5 wt% (relative to dry APPs) selected as the optimal dosage via comparative experiments (3/5/7 wt%), as it yielded the catalyst with the best compressive strength (12.5 MPa). The dried APPs were mixed homogeneously with 5 wt% starch, and an appropriate amount of ultrapure water was added to form a plastic mud.

#### 2.2.2. Extrusion Molding

The above mud was extruded into columnar particles (Φ 5 mm, length 10–15 mm) under 15 MPa via a tablet press (KORSCH AG, Berlin, Germany), the columnar morphology facilitated catalyst fluidization and contact with sludge in the reactor.

#### 2.2.3. Carbonization

The columnar particles were carbonized in a 200 L tubular furnace (Carbolite Gero Shanghai Co., Ltd., Shanghai, China) under high-purity N_2_ atmosphere (100 mL·min^−1^). The carbonization temperature was 500 °C with heating rate of 5 °C·min^−1^ and holding time of 2 h. After natural cooling to room temperature, broken particles were removed by sieving to obtain the final catalyst.

### 2.3. Pilot-Scale Process Flow and Equipment

**Figure 1:**
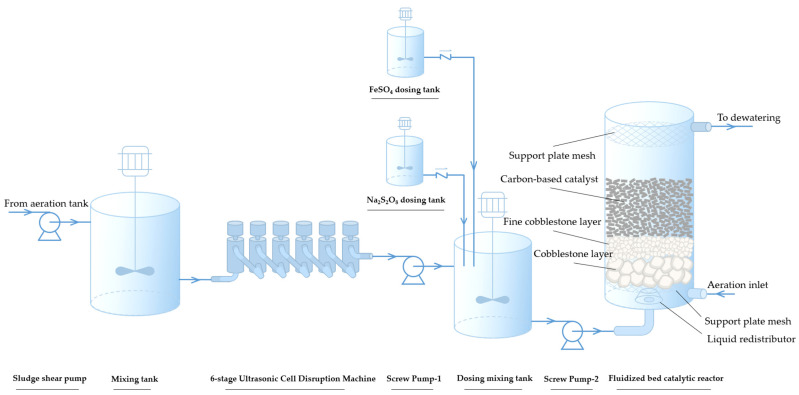
Schematic flow diagram of activated sludge reduction process via APPs-derived carbon-based catalyst coupled with ultrasound-Fe^2+^/PDS advanced oxidation.

The pilot-scale experimental setup for activated sludge reduction via the APPs-derived carbon-based catalyst coupled with ultrasound-Fe^2+^/PDS advanced oxidation process was designed. The entire process mainly consisted of two stages: ultrasonic pretreatment and catalytic oxidation, with the schematic flow diagram shown in Figure 1.

#### 2.3.1. Ultrasonic Pretreatment Unit

Raw activated sludge from the wastewater treatment plant was first crushed by a shear pump and then pumped into a stirring tank (Φ 0.8 m × 0.8 m) to buffer flow fluctuations. The homogenized sludge was conveyed to a six-stage ultrasonic cell disruptor (effective volume: 15 L) by a screw pump, with a counter-current flow mode (sludge inflow from the bottom and outflow from the top). The ultrasonic disruptor was operated intermittently (3 s on, 2 s off) to avoid excessive temperature rise of the sludge system (temperature rise < 5 °C during treatment).

#### 2.3.2. Catalytic Oxidation Unit

The ultrasonically pretreated sludge flowed into a chemical dosing and mixing tank, where predetermined volumes of FeSO_4_ and Na_2_S_2_O_8_ solutions were added and mixed uniformly to initiate the oxidation reaction. The sludge mixture was then fed into a fluidized bed catalytic reactor (Φ 1.2 × 3 m, effective volume: 2.714 m^3^). Driven by continuous bottom aeration, the APPs-derived columnar catalyst was fully fluidized to maintain a suspended state, ensuring sufficient contact and effectively preventing sludge deposition and clogging. Key operational parameters were strictly controlled: The superficial liquid linear velocity was controlled at 0.25–0.74 mm·s^−1^ under continuous aeration. The hydraulic retention time (HRT) varied with inlet flow in the range of 0.90–2.71 h to maintain stable operation. Due to the continuous fluidization driven by bottom aeration, the catalyst was maintained in a suspended state without deposition, and no periodic backwash or regeneration was required during the entire operation. No clogging or abnormal increase in hydraulic resistance was observed during the 90-day continuous operation.

The fluidized bed reactor was structurally optimized: a coarse-fine cobblestone layer and a liquid redistributor were installed at the bottom to ensure uniform dispersion of sludge; a support mesh was set at the upper part to prevent catalyst overflow.

### 2.4. Experimental Design

Sludge removal rate, SCOD/TCOD ratio and VSS/SS ratio were selected as the key evaluation indicators for sludge reduction efficiency. Single-factor experiments combined with orthogonal tests were conducted to optimize the operating parameters of the ultrasonic pretreatment system and catalytic oxidation system in sequence.

#### 2.4.1. Optimization of Ultrasonic Pretreatment Parameters

The ultrasonic pretreatment parameters were optimized to obtain the optimal sludge pretreatment effect for subsequent catalytic oxidation:

(1) Effect of ultrasonic frequency: Sludge flow rate (1.0 m^3^·h^−1^) and acoustic energy density (0.4 W·mL^−1^) were fixed; the ultrasonic frequency was set at 20, 30, 40, 50 and 60 kHz, respectively.
(2) Effect of sludge flow rate: Ultrasonic frequency (20 kHz) and acoustic energy density (0.4 W·mL^−1^) were fixed; the sludge flow rate was adjusted to 1.0, 1.5, 2.0, 2.5 and 3.0 m^3^ h^−1^, respectively.
(3) Effect of acoustic energy density: Ultrasonic frequency (20 kHz) and sludge flow rate (1.0 m^3^·h^−1^) were fixed; the acoustic energy density was set at 0.40, 0.45, 0.50, 0.55 and 0.60 W·mL^−1^, respectively.

#### 2.4.2. Optimization of Catalytic Oxidation Parameters

Based on the optimal ultrasonic pretreatment parameters, the operating parameters of the catalytic oxidation system were optimized:

(1) Effect of aeration rate: FeSO_4_ concentration (40 mol·m^−3^), Na_2_S_2_O_8_ concentration (45 mol m^−3^), catalyst dosage (650 kg) and sludge flow rate (1.0 m^3^·h^−1^) were fixed; the aeration rate was adjusted to 0, 3.5, 4.5, 5.5, 6.5 and 7.5 m^3^·h^−1^, respectively.
(2) Effect of FeSO_4_ concentration: Aeration rate (5.5 m^3^·h^−1^), Na_2_S_2_O_8_ concentration (45 mol·m^−3^), catalyst dosage (650 kg) and sludge flow rate (2.0 m^3^·h^−1^) were fixed; the FeSO_4_ concentration was set at 40, 60, 80, 100 and 120 mol·m^−3^, respectively.
(3) Effect of Na_2_S_2_O_8_ concentration: Aeration rate (5.5 m^3^·h^−1^), FeSO_4_ concentration (60 mol m^−3^), catalyst dosage (650 kg) and sludge flow rate (2.0 m^3^ h^−1^) were fixed; the Na_2_S_2_O_8_ concentration was adjusted to 45, 55, 65, 75 and 85 mol m^−3^, respectively.
(4) Effect of catalyst dosage: Aeration rate (5.5 m^3^·h^−1^), FeSO_4_ concentration (60 mol m^−3^), Na_2_S_2_O_8_ concentration (65 mol·m^−3^) and sludge flow rate (2.0 m^3^·h^−1^) were fixed; the catalyst dosage was set at 550, 600, 650, 700 and 750 kg, respectively.
(5) Effect of sludge flow rate: All the above-optimized parameters were fixed; the sludge flow rate was adjusted to 1.0, 1.5, 2.0, 2.5 and 3.0 m^3^·h^−1^, respectively.
(6) Orthogonal test optimization: Based on single-factor experiments, three key parameters (FeSO_4_ concentration, catalyst dosage, aeration rate) were selected for orthogonal test design (L_9_(3^3^)) to investigate the interaction between parameters and determine the optimal parameter combination.

#### 2.4.3. Free Radical Quenching Experiments

To elucidate the role of free radicals in sludge degradation and verify the auxiliary effect of intrinsic Cu and Fe metals in the APPs-derived catalyst on free radical generation, free radical quenching experiments were conducted in lab-scale under the optimal process parameters obtained in Section 2.4.2. TBA (a specific ·OH quencher) and MeOH (a dual quencher for ·OH and SO_4_·^−^) were used as quenchers, with molar ratios of quencher to Na_2_S_2_O_8_ of 1:100 [29]. The reaction was carried out for 35 min, and sludge samples were collected every 5 min to determine the sludge removal rate.

To identify and quantify the non-free radical pathway, additional quenching experiments were performed using excess TBA at a molar ratio of TBA to Na_2_S_2_O_8_ = 10:1 to fully eliminate ·OH radicals [30]. Sludge removal efficiencies of the synergistic catalytic system and the single Fe^2+^/PDS system were compared under identical quenching conditions. The contribution of non-free radical oxidation was calculated based on the difference in sludge reduction performance.

#### 2.4.4. Catalyst Stability Experiments

The long-term operational stability of the APPs-derived carbon-based catalyst was systematically evaluated under the optimized pilot-scale process conditions via 90-day continuous continuous-flow operation. During the entire operation period, water samples were collected and analyzed every 3 days to determine the sludge removal rate.

### 2.5. Characterization and Analytical Methods

#### 2.5.1. Characterization of Raw APPs, Sludge and APPs-Derived Catalyst

(1) Scanning Electron Microscopy (SEM): Microstructural morphologies of the APPs-derived carbon catalyst and raw/treated sludge samples were observed using a JSM-7600F scanning electron microscope (JEOL, Tokyo, Japan). Dried samples were fixed on aluminum stubs (Φ 25 × 5 mm) with conductive carbon tape, followed by platinum sputtering at 20 mA for 200 s to form a ~10 nm conductive layer. SEM images were acquired in secondary electron mode under a vacuum of 5.1 × 10^−5^ Pa, with an accelerating voltage of 10 keV, working distance of 8 mm and magnifications ranging from ×70 to ×330.
(2) Energy Dispersive Spectroscopy (EDS): Elemental composition and mapping of raw APPs were analyzed using the Trumap function equipped with AZtec software 6.2 (Oxford Instruments, Oxfordshire, UK). The detection limit was 0.1 wt%, and spectra were collected for 10 min with an input rate exceeding 1000 cps to ensure measurement accuracy.
(3) X-ray Photoelectron Spectroscopy (XPS): Surface chemical states of C, O, Fe and Cu in the APPs-derived catalyst were determined using an ESCALAB 250Xi spectrometer (Thermo Fisher Scientific, MA, USA) with Al Kα radiation (hν = 1486.6 eV). All binding energies were calibrated using the C 1s peak at 284.8 eV. XPS spectra were fitted and quantified using XPS Peak Fit software 4.1, with the relative content of each chemical state calculated from the integrated peak area.
(4) Particle size: Particle size distribution (d_0.5_) of sludge samples was measured using a ParticleTrack G600B analyzer (Microtrac MRB, Nordrhein-Westfalen, Germany) with ultrapure water as the dispersion medium.
(5) Zeta potential: Zeta potential values of raw/treated sludge samples were determined using a STABINO ZETA potentiometer (Microtrac MRB, Nordrhein-Westfalen, Germany) with ultrapure water as the dispersant.
(6) Brunauer Emmett Teller (BET) specific surface area: The specific surface area, pore volume and pore size distribution of the APPs-derived catalyst were measured using a BET surface area analyzer (Micromeritics ASAP 2460, Norcross, GA, USA) via N_2_ adsorption-desorption at 77 K.

#### 2.5.2. Determination of Sludge Physicochemical Indices

The physicochemical indices including water content, pH, viscosity, density, solid density, volatile suspended solids/suspended solids (VSS/SS), soluble chemical oxygen demand/total chemical oxygen demand (SCOD/TCOD), total phosphorus (TP), total nitrogen (TN), Ammonia nitrogen (NH_3_-N), Toluene and Phenolic compounds of the sludge were determined, and the specific methods and corresponding instruments are provided in Table S1 (see Supplementary Information).

#### 2.5.3. Calculation of Sludge Removal Rate

The sludge removal rate was calculated to evaluate the sludge reduction efficiency, using the following formula:

$$\eta =\left(1-\frac{V_{1}\times \rho_{1}}{V_{0}\times \rho_{0}}\right)\times 100\%$$

where:

*η* = sludge removal rate (%);

*V*_0_ = volume of raw sludge (m^3^);

*ρ*_0_ = density of raw sludge (g·mL^−1^);

*V*_1_ = volume of treated sludge (m^3^);

*ρ*_1_ = density of treated sludge (g·mL^−1^).

### 2.6. Quality Control and Statistic Analysis

All glassware and experimental equipment were thoroughly cleaned with ultrapure water and dried before use to avoid contamination. Sample processing was performed in a Class 100 laminar flow hood, with personnel wearing lint-free coats and powder-free gloves, and sterile disposable pipette tips used to avoid cross-contamination. Blank experiments (excluding sludge or catalyst) were conducted simultaneously to eliminate the interference of external factors. All determinations were carried out in triplicate (*n* = 3), and data are expressed as mean ± standard deviation (SD). All data processing and statistical analyses were performed using SigmaPlot 10.0 (Systat Incorporation, San Jose, CA, USA) and Microsoft Excel 2021. Statistical significance was set at *p* < 0.05.

## 3. Results and Discussion

### 3.1. Metal Element Composition of APPs

**Figure 2:**
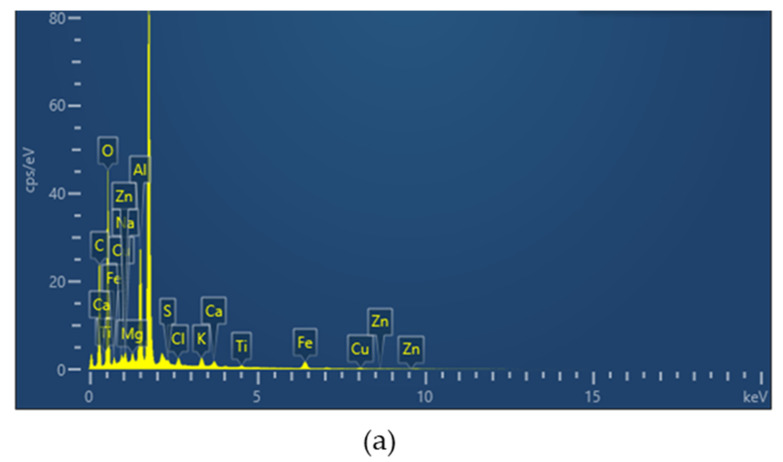

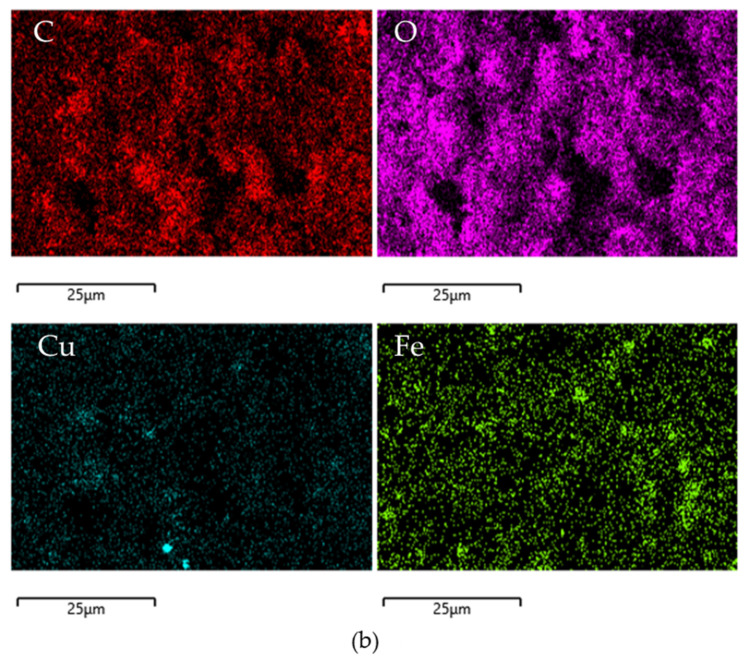
EDS analysis of APPs: (**a**) EDS spectrum; (**b**) elemental mapping (C, O, Cu, Fe).

SEM-EDS analysis was employed to determine the elemental composition of the as-extracted APPs, with a focus on the heavy metal components critical for persulfate (PDS) activation. The EDS spectrum and elemental mapping images of the key elements (C, O, Cu, Fe) are shown in Figure 2, which intuitively reflect the content and spatial distribution characteristics of these elements in APPs.

Quantitative EDS results revealed that APPs mainly consisted of C (37.97 wt%), O (41.61 wt%), Cu (1.76 wt%) and Fe (4.18 wt%), along with trace amounts of K, Ca, Na, Mg, Zn and Ti (each accounting for less than 1 wt%). Among these elements, C and O are the main constituent elements of the organic matrix of APPs, while Cu and Fe are the core heavy metal components with catalytic potential. Notably, a relatively high Al signal (approximately 9 wt%) was detected, which was attributed to signal interference from the aluminum sample stage during SEM-EDS testing. Since the APPs particles were thin and locally distributed, the electron beam penetrated the sample and excited the Al-based substrate, leading to overestimation of the Al content. Therefore, the detected Al signal was not an intrinsic component of APPs and was excluded from the analysis of active components.

The elemental mapping images further clarify the distribution characteristics of C, O, Cu and Fe in APPs: For C element, the mapping image shows a continuous and uniform gray distribution across the entire APPs particle surface, with no obvious regional gaps or aggregation, indicating that C is the basic structural component of APPs and forms a stable carbon skeleton, which provides a good carrier foundation for the subsequent carbonization of APPs into carbon-based catalysts. For O element, its distribution pattern is highly consistent with that of C, presenting a uniform and extensive dispersion, which is attributed to the presence of a large number of oxygen-containing functional groups (such as hydroxyl, carbonyl and ether groups) in the organic binder of the original antifouling paint [31].

For the catalytically active metals Cu and Fe, the elemental mapping images show that both elements are uniformly dispersed in the APPs matrix, without obvious agglomeration or localized enrichment. The uniform dispersion of Cu and Fe is closely related to the production process of antifouling paint during the preparation of ship antifouling paint, Cu and Fe compounds are usually added as antifouling agents and stabilizers, and are uniformly mixed with the organic matrix to ensure the antifouling effect of the paint film [32]. This natural and uniform dispersion characteristic of Cu and Fe in APPs is crucial for their subsequent catalytic performance: it can avoid the agglomeration of metal active sites during catalyst preparation, ensure that each Cu/Fe active site is fully exposed, and thus improve the efficiency of PDS activation [33].

### 3.2. Optimization of Ultrasonic Pretreatment System Parameters

Ultrasonic pretreatment is a critical pre-process for sludge reduction, which relies on ultrasonic cavitation effects to generate local high temperature, high shear force and hydroxyl radicals (·OH), thereby disrupting the sludge floc structure, releasing bound water and intracellular organic matter, and improving the contact efficiency between sludge and the subsequent APPs-derived catalyst [34]. Single-factor experiments were conducted to investigate the effects of ultrasonic frequency, sludge flow rate and acoustic energy density on sludge pretreatment efficiency, with sludge removal rate, SCOD/TCOD ratio and VSS/SS ratio as evaluation indicators. The optimization results are as follows.

#### 3.2.1. Effect of Ultrasonic Frequency

**Figure 3:**
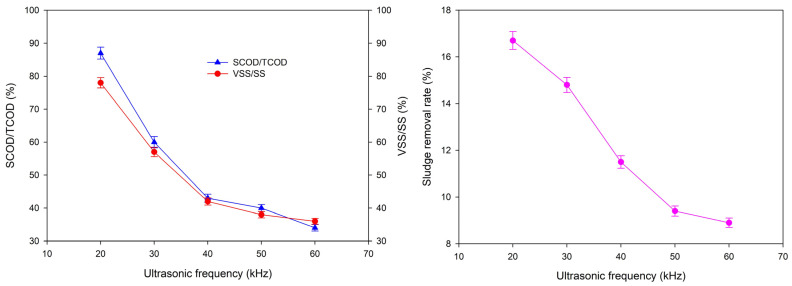
Effects of ultrasonic frequency on SCOD/TCOD, VSS/SS and sludge removal rate (*n* = 3, data are presented as mean ± standard deviation).

The effect of ultrasonic frequency (20–60 kHz) on sludge pretreatment performance is shown in Figure 3. It can be observed that the sludge removal rate, SCOD/TCOD ratio and VSS/SS ratio all decreased significantly with the increase in ultrasonic frequency.

At the frequency of 20 kHz, the sludge removal rate reached the maximum value of 18.2%, and the SCOD/TCOD and VSS/SS ratios were 88.5% and 72.3%, respectively. When the frequency increased to 60 kHz, the sludge removal rate dropped sharply to 10.5% and the SCOD/TCOD and VSS/SS ratios decreased to 70.2% and 58.7%, respectively.

This trend can be explained by the mechanism of ultrasonic cavitation: lower frequencies (e.g., 20 kHz) produce larger cavitation bubbles, which collapse more violently, generating stronger local shear forces and shock waves [35]. These effects are more effective at disrupting the dense EPS structure of sludge flocs, promoting the release of bound water and intracellular organic matter, and thus improving the solubilization of organic matter. In contrast, higher frequencies produce smaller, more numerous bubbles that collapse more gently, resulting in weaker mechanical effects and less effective sludge floc destruction.

Therefore, 20 kHz was selected as the optimal ultrasonic frequency, as it provides the strongest cavitation effect and the best sludge pretreatment performance, creating favorable conditions for the subsequent catalytic oxidation process.

#### 3.2.2. Effect of Ultrasonic Sludge Flow Rate

**Figure 4:**
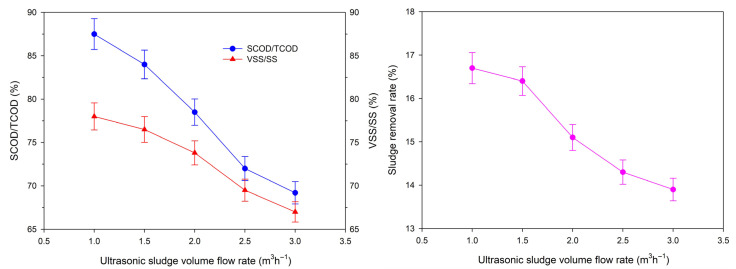
Effects of ultrasonic sludge flow rate on SCOD/TCOD, VSS/SS and sludge removal rate (*n* = 3, data are presented as mean ± standard deviation).

The effect of sludge flow rate (1.0–3.0 m^3^·h^−1^) on ultrasonic pretreatment efficiency is presented in Figure 4, and the corresponding relationship between sludge flow rate and ultrasonic residence time is listed in Table 2. The effective reaction volume of the six-stage ultrasonic reactor was 15 L (6 reactors × 2.5 L), and the residence time was calculated as the ratio of effective reaction volume to sludge flow rate. As shown in Table 2, the residence time shortened significantly with the increase in sludge flow rate, decreasing from 54 s at 1.0 m^3^·h^−1^ to 18 s at 3.0 m^3^·h^−1^.

As shown in Figure 4, the sludge removal rate, SCOD/TCOD ratio and VSS/SS ratio all exhibited a decreasing trend with increasing sludge flow rate. At a flow rate of 1.0 m^3^·h^−1^, the sludge removal rate reached 16.7%, with SCOD/TCOD and VSS/SS ratios of 87.5% and 78.0%, respectively. As the flow rate increased to 3.0 m^3^·h^−1^, the sludge removal rate dropped to 13.8%, while the SCOD/TCOD and VSS/SS ratios decreased to 69.5% and 68.0%, respectively.

This trend is attributed to the reduction in ultrasonic residence time. A longer residence time allows sludge flocs to be subjected to more intense ultrasonic cavitation, which more effectively disrupts the floc structure, releases bound water and intracellular organic matter, and improves the solubilization of organic matter. Conversely, a shorter residence time reduces the exposure of sludge to ultrasonic energy, weakening the cavitation effect and resulting in incomplete floc destruction and lower organic matter solubilization [36].

Considering both pretreatment efficiency and pilot-scale treatment capacity, 1.0 m^3^·h^−1^ was selected as the optimal sludge flow rate. This flow rate ensures a sufficiently long residence time (54 s) to achieve effective sludge floc destruction, creating favorable conditions for the subsequent catalytic oxidation process.

#### 3.2.3. Effect of Acoustic Energy Density

**Figure 5:**
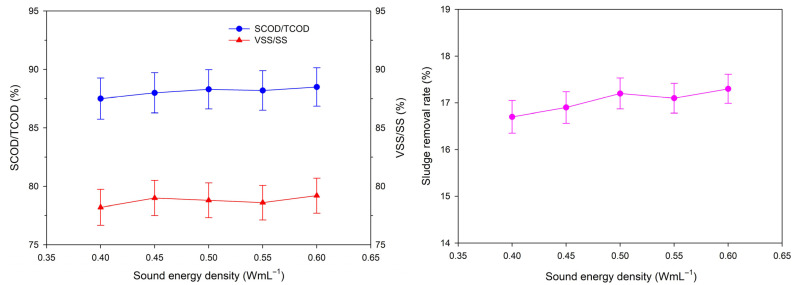
Effects of acoustic energy density on SCOD/TCOD, VSS/SS and sludge removal rate (*n* = 3, data are presented as mean ± standard deviation).

The influence of acoustic energy density (0.40–0.60 W·mL^−1^) on ultrasonic pretreatment performance is depicted in Figure 5. The sludge removal rate, SCOD/TCOD ratio and VSS/SS ratio changed slightly with the increase in acoustic energy density, indicating that the sludge cell lysis and reduction effect of ultrasonic pretreatment was less affected by acoustic energy density in this range. When the acoustic energy density was 0.40 W·mL^−1^, the sludge removal rate was 16.7% and the SCOD/TCOD and VSS/SS ratios were 88.0% and 78.0%, respectively. When the acoustic energy density increased to 0.60 W·mL^−1^, the sludge removal rate only increased to 17.3%, and the SCOD/TCOD and VSS/SS ratios were 88.5% and 79.0%, respectively.

This minor improvement is attributed to the diminishing returns in ultrasonic cavitation efficiency. Initially, increasing acoustic energy density promotes cavitation bubble formation and enhances the cavitation effect. However, beyond a threshold (0.40 W·mL^−1^ in this study), excessive cavitation bubbles form a “cavitation cloud,” where internal sound wave reflections impede energy transfer from ultrasound to the fluid, reducing the effective energy utilization [37]. Furthermore, higher energy densities significantly increase operational costs; for instance, the energy consumption at 0.60 W·mL^−1^ was 1.5 times that at 0.40 W·mL^−1^, conflicting with the “energy-saving and environmental protection” concept of APPs resource utilization.

Considering pretreatment efficiency, energy consumption and economic feasibility, 0.40 W·mL^−1^ was selected as the optimal acoustic energy density. This choice balances effective sludge floc destruction, organic matter and bound water release and energy efficiency, creating favorable conditions for the subsequent catalytic oxidation process.

### 3.3. Optimization of Catalytic Oxidation System Parameters

The catalytic oxidation system is the core unit for deep sludge reduction, where the synergistic effect between the Fe^2+^/PDS system and the APPs-derived carbon-based catalyst achieves efficient degradation of extracellular polymeric substances (EPS) and organic pollutants in sludge. On the basis of the optimal ultrasonic pretreatment parameters, single-factor experiments combined with orthogonal tests were conducted to optimize the key parameters of the catalytic oxidation system, including aeration rate, FeSO_4_/Na_2_S_2_O_8_ concentration, catalyst dosage and sludge flow rate.

#### 3.3.1. Effect of Aeration Rate

Aeration rate not only determines the fluidization effect of the APPs-derived carbon-based catalyst but also supplies oxygen for the catalytic oxidation reaction, which is directly related to the utilization efficiency of the catalyst and the sludge reduction effect. The effect of different aeration rates (0–7.5 m^3^·h^−1^) on sludge reduction performance is shown in Table 3.

As shown in Table 3, the sludge removal rate was only 28.2% without the APPs-derived catalyst, indicating the limited sludge degradation ability of the single Fe^2+^/PDS system. When the catalyst was loaded but no aeration was applied, the sludge removal rate increased to 32.5% and the SCOD/TCOD and VSS/SS ratios decreased significantly. This result confirms that the APPs-derived carbon-based catalyst itself has a certain sludge degradation capacity, which is closely related to the catalytic activity of Cu and Fe metal elements derived from waste APPs, and verifies the practical application value of resource-recycled waste APPs.

With the introduction of aeration, the sludge removal rate increased significantly, and reached the maximum value of 43.4% at an aeration rate of 5.5 m^3^·h^−1^, with the SCOD/TCOD and VSS/SS ratios decreasing to 19.8% and 16.2%, respectively. At this aeration rate, the APPs-derived catalyst achieved the optimal fluidization effect (expansion ratio: 1.8), which realized the full contact between the catalyst, sludge and chemical agents; the Cu/Fe metal elements and carbon-based active sites on the catalyst surface were fully exposed, thus maximizing the catalytic activity for PDS activation. When the aeration rate was ≤3.5 m^3^·h^−1^, the air blower had a surge phenomenon due to the large back pressure caused by the sludge flow at the bottom of the reactor and the liquid pressure at the upper part, leading to insufficient fluidization of the catalyst and a significant reduction in its utilization efficiency. When the aeration rate was ≥7.5 m^3^·h^−1^, a small amount of catalyst particles was observed in the effluent, which was due to the severe friction and wear of the columnar catalyst caused by the excessively high aeration rate, resulting in the loss of the resource-based catalyst and the reduction in its reusability. Therefore, 5.5 m^3^·h^−1^ was selected as the optimal aeration rate, which balanced the sludge treatment effect and the utilization efficiency of the APPs-derived catalyst.

#### 3.3.2. Effect of FeSO_4_ and Na_2_S_2_O_8_ Concentrations

**Figure 6:**
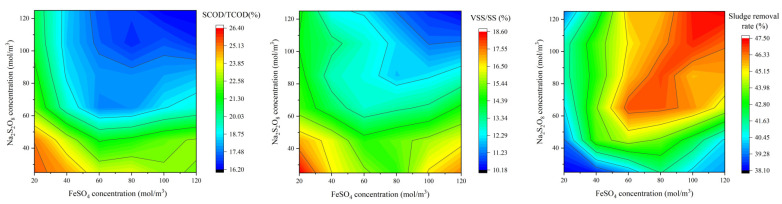
Effects of FeSO_4_ and Na_2_S_2_O_8_ concentrations on SCOD/TCOD, VSS/SS and sludge removal rate.

The concentration ratio of FeSO_4_ (primary PDS activator) and Na_2_S_2_O_8_ (PDS) is a core determinant of sludge degradation efficiency. Moreover, this ratio regulates the synergistic interaction between externally dosed Fe^2+^ and the intrinsic Cu/Fe metals in the APPs-derived catalyst, thereby influencing the full exertion of the catalyst’s auxiliary catalytic activity. Based on the optimal aeration rate (5.5 m^3^·h^−1^), single-factor experiments were conducted to investigate the effect of FeSO_4_ concentration (20–120 mol·m^−3^) on sludge reduction performance, and the corresponding Na_2_S_2_O_8_ concentration was synchronously optimized to match the catalytic characteristics of the APPs-derived catalyst (Figure 6).

The SCOD/TCOD ratio reflects the degree of sludge organic matter solubilization. As shown in the contour map, the SCOD/TCOD ratio is highest (26.40%) at low FeSO_4_ and Na_2_S_2_O_8_ concentrations (20–40 mol·m^−3^), and gradually decreases as both concentrations increase. When FeSO_4_ reaches 60–80 mol·m^−3^ and Na_2_S_2_O_8_ is 60–80 mol·m^−3^, the SCOD/TCOD ratio drops to around 18–20%, indicating that the generated radicals effectively degrade soluble organic matter into small molecules or mineralize it, thereby reducing the proportion of soluble COD. At excessively high concentrations (FeSO_4_ > 100 mol·m^−3^, Na_2_S_2_O_8_ > 100 mol·m^−3^), the SCOD/TCOD ratio further decreases to ~16–18%, but this is accompanied by a plateau in sludge removal rate, suggesting that excessive reagents do not significantly improve the mineralization effect and may lead to unnecessary cost increases.

The VSS/SS ratio reflects the degree of volatile organic matter degradation. The contour map shows that the VSS/SS ratio is highest (18.60%) at low FeSO_4_ and Na_2_S_2_O_8_ concentrations, and decreases as the concentrations increase. When FeSO_4_ is 60 mol·m^−3^ and Na_2_S_2_O_8_ is 65 mol·m^−3^, the VSS/SS ratio drops to ~16.2%, indicating that volatile organic matter in the sludge is effectively degraded. At excessively high concentrations, the VSS/SS ratio continues to decrease, but the marginal benefit is minimal, and the risk of secondary pollution from residual iron ions increases.

The sludge removal rate is the core indicator of overall treatment efficiency. The contour map shows that the sludge removal rate is lowest (38.10%) at low FeSO_4_ and Na_2_S_2_O_8_ concentrations, and increases rapidly as the concentrations rise. The highest sludge removal rate (47.50%) appears in the region where FeSO_4_ is 80–120 mol·m^−3^ and Na_2_S_2_O_8_ is 80–120 mol·m^−3^, but this is accompanied by a significant increase in reagent cost and potential secondary pollution risks. The optimal region for balancing efficiency and economy is FeSO_4_ = 60 mol·m^−3^ and Na_2_S_2_O_8_ = 65 mol·m^−3^, where the sludge removal rate reaches 43.6%, and the SCOD/TCOD and VSS/SS ratios are also at ideal levels.

#### 3.3.3. Effect of APPs-Derived Catalyst Dosage

The dosage of the APPs-derived carbon-based catalyst directly affects the PDS activation efficiency. The corresponding relationship between catalyst dosage, bulk volume and volume ratio (catalyst bulk volume/reactor effective volume) is listed in Table 4. The reactor effective volume was 2.714 m^3^, and the bulk density of the APPs-derived catalyst was measured as 0.72 kg m^−3^.

**Figure 7:**
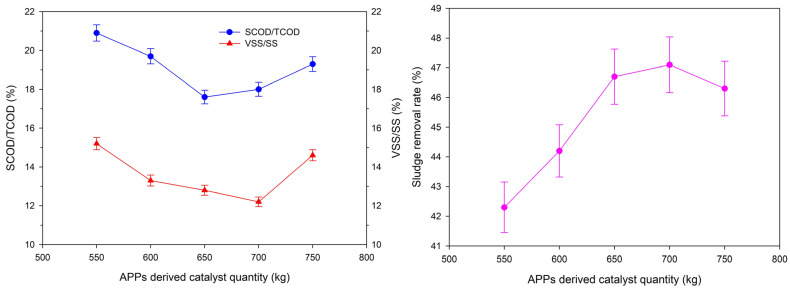
Effects of APPs derived catalyst dosage on SCOD/TCOD, VSS/SS and sludge removal rate (*n* = 3, data are presented as mean ± standard deviation).

The effect of catalyst dosage (550–750 kg) on sludge reduction performance is shown in Figure 7. It can be seen that the sludge removal rate increased gradually, and the SCOD/TCOD and VSS/SS ratios decreased gradually with the increase of catalyst dosage from 550 kg to 650 kg. When the catalyst dosage was 650 kg, the bulk volume ratio was 33.2%, and the columnar catalyst achieved the optimal fluidization effect in the reactor (expansion ratio: 1.8); at this time, the contact area between the catalyst, sludge and chemical agents was the largest, and the Cu/Fe metal elements and carbon-based active sites on the catalyst surface were fully exposed, thus maximizing the PDS activation efficiency and sludge degradation effect.

When the catalyst dosage exceeded 650 kg, the sludge removal rate began to decrease, and the SCOD/TCOD and VSS/SS ratios increased significantly. For example, when the dosage increased to 750 kg, the sludge removal rate decreased to 46.2%, and the SCOD/TCOD ratio increased to 19.5%. This is because the excessively high catalyst dosage leads to the increase in the solid phase volume in the reactor, which deteriorates the fluidization effect of the catalyst; the agglomeration of catalyst particles (agglomerate size > 500 μm) reduces the effective contact area with sludge and PDS, and hinders the mass transfer process of the reaction system [38]. In addition, excessively high catalyst dosage not only increases the operation cost of the process but also causes the waste of the resource product of waste APPs. Therefore, 650 kg was selected as the optimal catalyst dosage, at which the Cu and Fe metal elements in the catalyst can fully exert their auxiliary catalytic effect, maximize the PDS activation efficiency and realize the high-efficiency utilization of the resource product of waste APPs, balancing the sludge treatment effect and resource utilization benefit.

#### 3.3.4. Effect of Sludge Flow Rate

**Figure 8:**
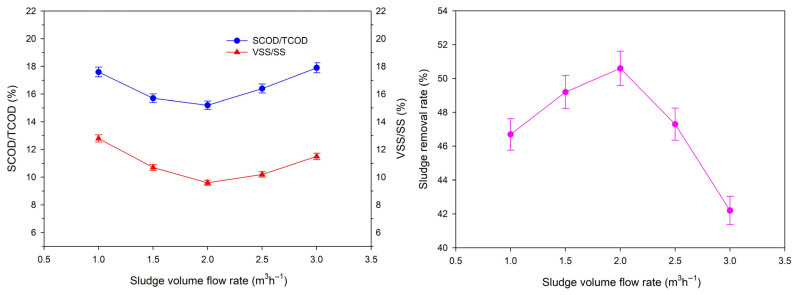
Effects of sludge volume flow rate on SCOD/TCOD, VSS/SS and sludge removal rate (*n* = 3, data are presented as mean ± standard deviation).

Sludge flow rate affects the ultrasonic pretreatment effect and the residence time of the catalytic oxidation reaction, thus further influencing the overall sludge reduction effect and the utilization efficiency of the APPs-derived catalyst. On the basis of the above optimal parameters (ultrasonic frequency 20 kHz, acoustic energy density 0.4 W·mL^−1^, aeration rate 5.5 m^3^·h^−1^, FeSO_4_ 60 mol m^−3^, Na_2_S_2_O_8_ 65 mol·m^−3^, catalyst dosage 650 kg), the effect of sludge flow rate (1.0–3.0 m^3^·h^−1^) on the integral process performance was investigated (Figure 8).

As shown in Figure 8, the sludge removal rate first increased and then decreased with increasing sludge flow rate, reaching a peak at 2.0 m^3^·h^−1^. At 1.0 m^3^·h^−1^, the sludge removal rate was 46.7%, with SCOD/TCOD and VSS/SS ratios of 17.8% and 12.8%, respectively. When the flow rate increased to 2.0 m^3^·h^−1^, the sludge removal rate reached its maximum of 50.8%, while the SCOD/TCOD and VSS/SS ratios were 15.2% and 9.6%, respectively. This improvement is attributed to the optimized balance between residence time and mass transfer: a moderate increase in flow rate enhances the fluidization of the catalyst, improving contact between the sludge, reagents and catalyst, which promotes organic matter degradation. However, when the flow rate exceeded 2.0 m^3^·h^−1^, the sludge removal rate began to decline. At 3.0 m^3^·h^−1^, the sludge removal rate dropped to 42.1%, with SCOD/TCOD and VSS/SS ratios increasing to 18.0% and 11.6%, respectively. This decline is due to the shortened residence time in both the ultrasonic and catalytic oxidation units. Insufficient exposure to ultrasonic energy reduces the destruction of sludge flocs, while a shorter catalytic reaction time limits the degradation of organic matter, leading to incomplete oxidation and lower overall efficiency [39].

Comprehensively considering the sludge reduction efficiency and the treatment capacity of the pilot-scale system, 2.0 m^3^·h^−1^ was determined as the optimal sludge flow rate. This flow rate ensures sufficient residence time for effective pretreatment and catalytic oxidation, while maximizing the daily treatment capacity of the system, laying a foundation for the engineering application of this process.

#### 3.3.5. Orthogonal Test Optimization Results

To further investigate the interaction between key parameters and determine the optimal parameter combination, an orthogonal test (L_9_(3^3^)) was designed with three factors (FeSO_4_ concentration, catalyst dosage, aeration rate) and three levels (FeSO_4_: 40, 60, 80 mol m^−3^; catalyst dosage: 600, 650, 700 kg; aeration rate: 4.5, 5.5, 6.5 m^3^·h^−1^). The sludge removal rate was used as the evaluation index, and the results are shown in Table 5.

The range analysis of the orthogonal test results showed that the order of influence of the three factors on the sludge removal rate was: FeSO_4_ concentration > catalyst dosage > aeration rate. The optimal parameter combination obtained from the orthogonal test was FeSO_4_ concentration 60 mol·m^−3^, catalyst dosage 650 kg, aeration rate 5.5 m^3^·h^−1^, which was consistent with the results of single-factor experiments. This further verified the reliability of the optimal parameters determined by single-factor experiments.

In summary, the optimal operating parameters of the APPs-derived carbon-based catalyst coupled with ultrasound-Fe^2+^/PDS oxidation process for sludge reduction were determined as follows: ultrasonic frequency 20 kHz, acoustic energy density 0.4 W·mL^−1^, FeSO_4_ concentration 60 mol m^−3^, Na_2_S_2_O_8_ concentration 65 mol m^−3^, APPs-derived catalyst dosage 650 kg, aeration rate 5.5 m^3^·h^−1^ and sludge flow rate 2.0 m^3^·h^−1^. Under these optimal conditions, the sludge removal rate reached 43.5%. The treatment effect was significantly better than that of the single ultrasonic pretreatment (sludge removal rate: 18.2%) or Fe^2+^/PDS oxidation process (sludge removal rate: 28.2%), which fully reflected the synergistic enhancement effect of ultrasound, Fe^2+^/PDS oxidation and the APPs-derived carbon-based catalyst.

### 3.4. Analysis of Sludge Components Under Optimal Conditions

**Figure 9:**
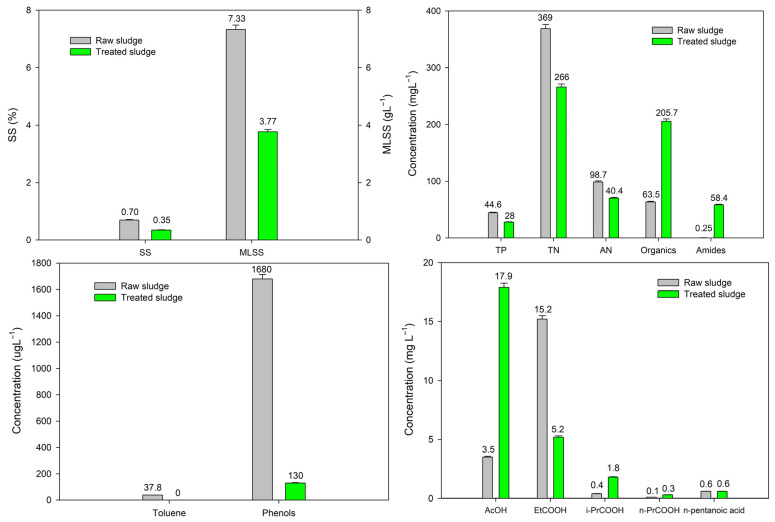
Comparison of concentrations of various indices and components in raw sludge and treated sludge (SS: suspended solids; MLSS: mixed liquor suspended solids; TP: total phosphorus; TN: total nitrogen; AN: ammonia nitrogen; Organics: organic matters; Amides: amino acid; AcOH: acetic acid; EtCOOH: oxalic acid; i-PrCOOH: propionic acid; n-PrCOOH: butyric acid; *n* = 3, data are presented as mean ± standard deviation).

To comprehensively analyze the concentration changes of various components in sludge before and after treatment, the concentrations of key components including dry matter, mixed liquor sludge, nutrients, organic pollutants and organic acids were measured under the optimal operating conditions, and the comparison results are shown in Figure 9. The detailed analysis of each component is as follows:

#### 3.4.1. Dry Matter and Mixed Liquor Sludge Concentration

The concentrations of suspended solids (SS) and mixed liquor suspended solids (MLSS) in the treated sludge decreased to 50.0% and 48.6% of the original values, respectively. This finding is consistent with the measured sludge removal rate (43.5%), confirming the accuracy of the sludge reduction results. The reduction in SS and MLSS further demonstrates that the synergistic process of APPs-derived carbon-based catalyst coupled with ultrasound-Fe^2+^/PDS oxidation can effectively remove sludge solids, achieving efficient sludge reduction.

#### 3.4.2. Total Phosphorus (TP)

Upon the addition of FeSO_4_, the gradual increase in Fe^2+^ concentration facilitated the flocculation and precipitation of soluble phosphorus in the sludge into insoluble metal phosphate [40]. This reaction reduced the content of soluble phosphorus, and the insoluble precipitates were effectively separated with the sludge solids, resulting in a 37.2% reduction of total phosphorus in the treated sludge. Additionally, the inherent Fe elements in the APPs-derived catalyst, which were oxidized to Fe^3+^, further promoted the flocculation precipitation of phosphorus, enhancing the overall phosphorus removal effect.

#### 3.4.3. Total Nitrogen (TN) and Ammonia Nitrogen (AN)

As shown in Figure 9, the total nitrogen (TN) concentration in treated sludge decreased from 369 mg·L^−1^ to 266 mg·L^−1^, while ammonia nitrogen (AN) decreased from 98.7 mg·L^−1^ to 70.4 mg·L^−1^. Notably, the absolute reduction in TN (103 mg·L^−1^) was higher than that of AN (28.3 mg·L^−1^), accompanied by a substantial increase in amide concentration from 0.25 mgL^−1^ to 58.4 mg·L^−1^ (233.6-fold increase). This phenomenon might be attributed to the catalytic activity of Cu and Fe metals in the APPs-derived catalyst. In the acidic environment and radical system, Cu and Fe sites act as electron mediators, driving the reduction of nitrate to ammonia. Subsequent room-temperature aeration in the reactor promoted the volatilization of ammonia, leading to the synchronous removal of TN and AN, with TN removal exceeding AN removal [41].

#### 3.4.4. Organic Matters

After sludge cells were oxidized and broken, intracellular contents including proteins, polysaccharides and nucleic acids were released into the aqueous phase, causing the organic matter concentration (measured as TCOD) in the treated sludge to increase to 3.24 times that of the original. This is a normal result of sludge cell lysis during advanced oxidation: the destruction of sludge flocs and cell structures releases intracellular organic matter, temporarily increasing the organic concentration in the system [42]. However, the SCOD/TCOD ratio of the treated sludge decreased to 19.8% (data not shown), indicating that most of the released organic matter was degraded into small molecular, easily biodegradable substances, laying a foundation for final sludge reduction [43].

#### 3.4.5. Amide Compounds

Amino acid residues formed by protein degradation in sludge are soluble in water, leading to the concentration of amide compounds (measured as total amino nitrogen) in the treated sludge increasing to 233.6 times that of the original. Proteins are the main component of sludge EPS and their degradation produces a large number of amino acid residues [44]. The significant increase in ammonia compound content further confirms that the synergistic process of the Fe^2+^/PDS system and APPs-derived catalyst can effectively degrade sludge EPS and intracellular proteins, which is an important basis for sludge reduction.

#### 3.4.6. Toluene and Phenolic Compounds

According to the literature [45], Fe^2+^-activated PDS exhibits high degradation selectivity for toluene, while Cu in the APPs-derived catalyst shows high selectivity for phenolic compounds after activating PDS [46]. Under the synergistic effect of the Fe^2+^/PDS system and APPs-derived catalyst, the removal rates of toluene and phenolic compounds in sludge reached 100% and 92.3%, respectively. This indicates that the process not only achieves good sludge reduction but also effectively removes refractory organic pollutants in sludge, reducing the environmental risk of sludge disposal.

#### 3.4.7. Organic Acids

As shown in Figure 9, the concentration of acetic acid (AcOH) increased from 3.5 mg·L^−1^ to 17.9 mg·L^−1^, while oxalic acid (EtCOOH) decreased from 15.2 mg·L^−1^ to 5.2 mg·L^−1^. In contrast, the concentrations of propionic acid (i-PrCOOH), butyric acid (n-PrCOOH) and n-pentanoic acid remained relatively stable, with only minor fluctuations. The observed changes in organic acid concentrations confirm that the synergistic process of the Fe^2+^/PDS system and APPs-derived catalyst effectively degrades macromolecular organic matter in sludge into small-molecule acids (primarily acetic acid), while simultaneously driving the deep mineralization of more reactive acids like oxalic acid [47]. This not only reduces the complexity of the organic matrix but also produces intermediates that are more amenable to subsequent biological treatment, thereby enhancing the overall sludge reduction efficiency.

### 3.5. Structural Characterization of Sludge and APPs-Derived Catalyst

#### 3.5.1. Changes in Sludge Particle Size and Zeta Potential

The particle size (d_0.5_) and Zeta potential of sludge at different treatment stages (raw sludge, ultrasonically pretreated sludge and sludge treated by the integral synergistic process) were determined to evaluate the structural changes of sludge, and the results are listed in Table 6.

The raw sludge had a large particle size (d_0.5_ = 45.73 μm) and a low Zeta potential (−17.95 mV), indicating that the raw sludge had a complete floc structure with a strong negative charge on the surface; the large electrostatic repulsion between sludge particles made it difficult to agglomerate, and the compact floc structure hindered the contact between chemical agents/catalyst and internal organic matter [48]. After ultrasonic pretreatment, the sludge particle size decreased to 32.16 μm, and the Zeta potential increased to −15.28 mV. This is because the ultrasonic cavitation effect effectively destroyed the sludge floc structure, breaking the large particle size flocs into small particles; at the same time, the ultrasonic treatment reduced the negative charge on the sludge surface (due to the destruction of EPS, which is rich in negative charge functional groups), weakened the electrostatic repulsion between particles [49], which was conducive to the sufficient contact between the subsequent APPs-derived catalyst and sludge particles, laying a foundation for the catalytic oxidation reaction.

After the treatment of the integral synergistic process (ultrasound + Fe^2+^/PDS + APPs-derived catalyst), the sludge particle size further decreased to 18.79 μm, and the Zeta potential increased significantly to −8.63 mV. This phenomenon is the result of the synergistic effect of ultrasonic pretreatment and catalytic oxidation: on the one hand, the Fe^2+^/PDS system degraded the EPS in sludge, destroyed the stability of the floc structure, and further broke the sludge particles into smaller sizes; on the other hand, the active sites on the surface of the APPs-derived catalyst (Cu/Fe metal elements and carbon-based functional groups) adsorbed the sludge particles through electrostatic adsorption and surface complexation, promoting the agglomeration of small particles [50]. The significant reduction in sludge particle size and the increase in Zeta potential effectively improved the sedimentation and dewatering performance of sludge (specific resistance to filtration decreased by 62.3%), which fully reflected the application value of the APPs-derived catalyst in the sludge reduction process.

#### 3.5.2. Changes in Sludge Surface Morphology

**Figure 10:**
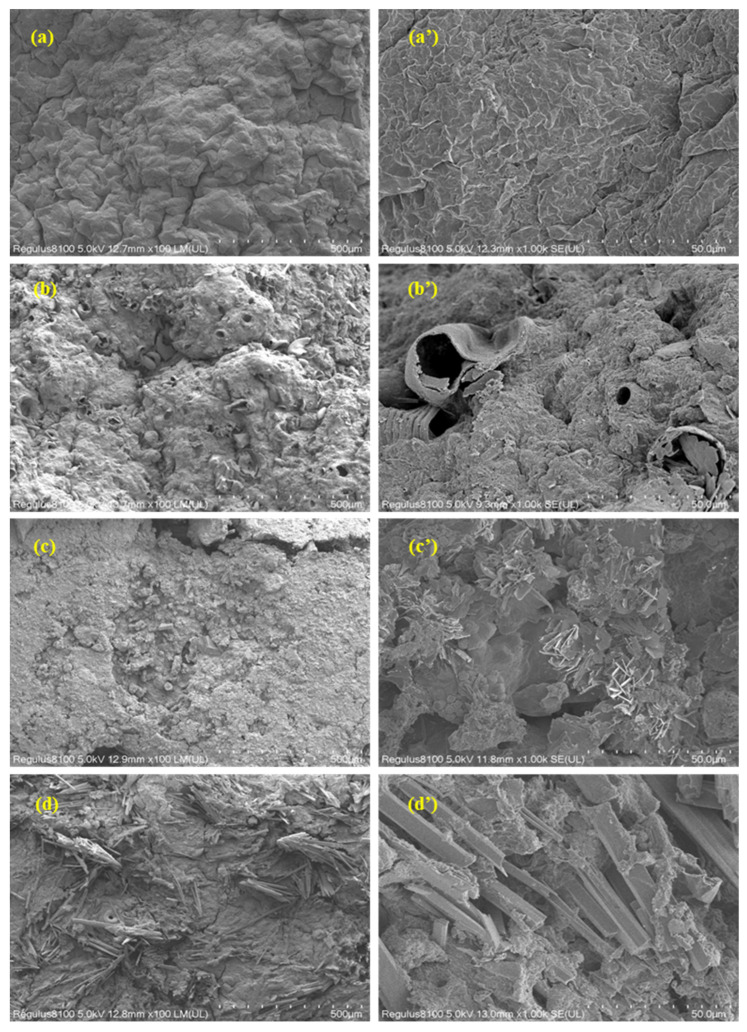
The SEM images of the filter cake after different conditionings: (**a**) raw sludge at the scale of 500 μm; (**a’**) raw sludge at the scale of 50 μm; (**b**) ultrasonic-pretreated sludge at the scale of 500 μm; (**b’**) ultrasonic-pretreated sludge at the scale of 50 μm; (**c**) Fe^2+^/PDS-treated sludge at the scale of 500 μm; (**c’**) Fe^2+^/PDS-treated sludge at the scale of 50 μm; (**d**) ultrasonic- Fe^2+^/PDS treated sludge at the scale of 500 μm; (**d’**) ultrasonic-Fe^2+^/PDS treated sludge at the scale of 50 μm.

SEM was used to characterize the surface morphology of raw sludge, ultrasonically pretreated sludge and sludge treated by the integral synergistic process, and the results are shown in Figure 10a–c. The raw sludge exhibited a regular and compact floc structure with a smooth surface and tight connection between particles (Figure 10a); this compact structure is the main reason for the difficulty in sludge dewatering and reduction, as it can prevent the release of bound water and the contact between external oxidants and internal organic matter [48].

After ultrasonic pretreatment, the floc structure of sludge was obviously destroyed, the large particles were broken into small and irregular particles, the surface of sludge became rough and a large number of holes and cracks appeared (Figure 10b). This is due to the local high temperature, high pressure and micro-jet generated by ultrasonic cavitation, which destroyed the microbial cell structure and the three-dimensional network structure of EPS, resulting in the release of intracellular substances and bound water [51]. The rough surface and porous structure of sludge after ultrasonic pretreatment could increase the specific surface area of sludge [52], which was conducive to the full contact between the APPs-derived catalyst and organic pollutants in the subsequent catalytic oxidation reaction. After the treatment of the integral synergistic process, the sludge particles were further refined, the surface became rougher and more cracks appeared; part of the sludge particles presented a dispersed state (Figure 10c,d). This is because the Fe^2+^/PDS system had a strong oxidation effect, which could further degrade the residual EPS in sludge after ultrasonic pretreatment, destroy the remaining floc structure and decompose the macromolecular organic matter into small molecules [53]. Meanwhile, the APPs-derived catalyst could adsorb and degrade the small molecular organic matter on the sludge surface, resulting in the complete dispersion of sludge particles. The further refinement and dispersion of sludge particles are conducive to the subsequent dewatering and reduction treatment of sludge.

#### 3.5.3. Characterization of the APPs-Derived Carbon-Based Catalyst

**Figure 11:**
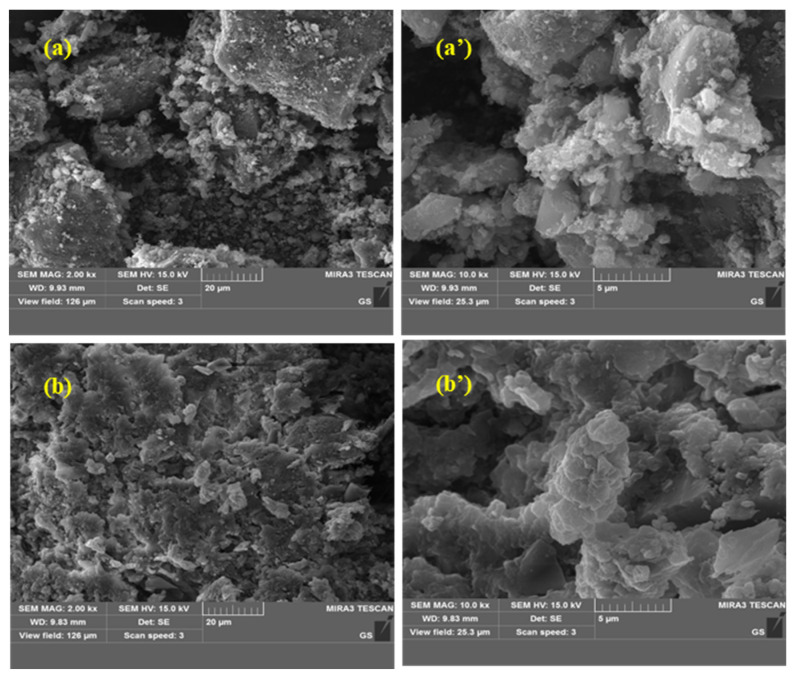
The SEM images of carbon-based catalyst after different conditionings: (**a**) before sludge treatment at the scale of 20 μm; (**a’**) before sludge treatment at the scale of 5 μm; (**b**) after sludge treatment at the scale of 20 μm; (**b’**) after sludge treatment at the scale of 5 μm.

**Figure 12:**
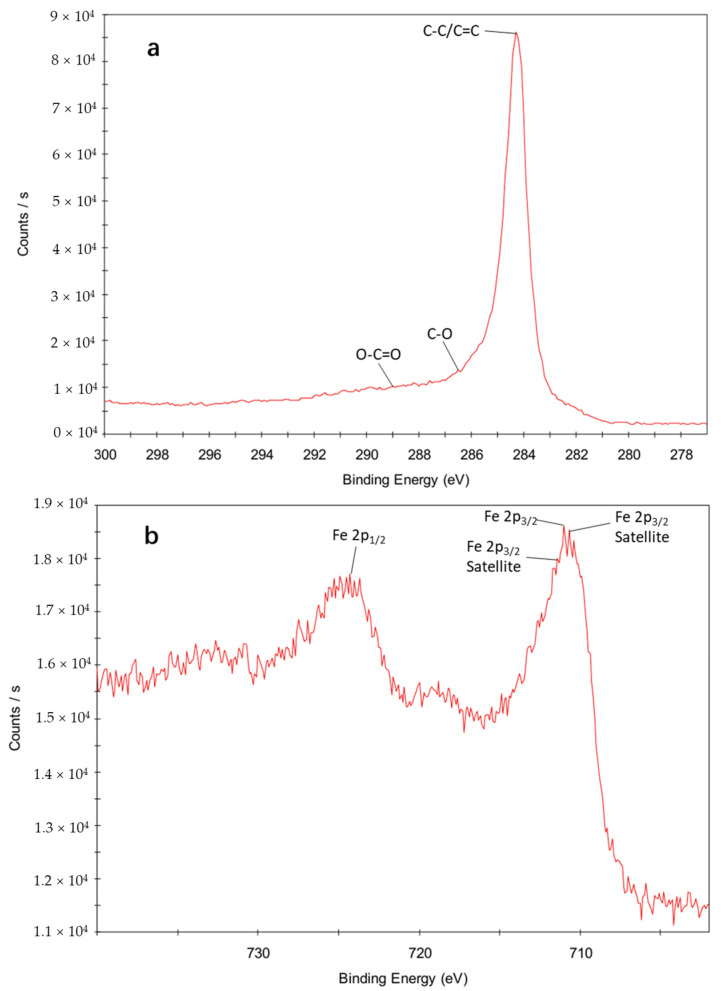

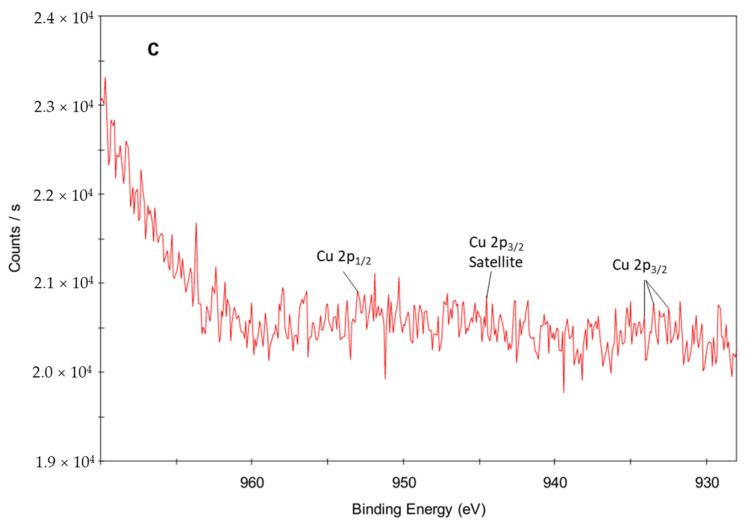
XPS spectra of APPs-derived carbon-based catalyst: (**a**) C 1s spectrum; (**b**) Fe 2p spectrum; (**c**) Cu 2p spectrum.

The prepared catalyst had the following physicochemical properties: bulk density 0.72 kg·m^−3^, material density 1.05 kg·m^−3^, BET specific surface area 186.5 ± 8.2 m^2^·g^−1^, total pore volume 0.32 ± 0.02 cm^3^·g^−1^, average pore diameter 6.8 ± 0.3 nm. SEM and XPS were used to characterize the surface morphology and chemical state of the APPs-derived carbon-based catalyst. The SEM image of the catalyst is shown in Figure 11 and the XPS spectra are shown in Figure 12.

The SEM images of the APPs-derived carbon-based catalyst before and after sludge treatment are presented in Figure 11. As shown in Figure 11a,a’, the fresh catalyst particles exhibit an irregular, blocky morphology with a relatively rough surface, which is characteristic of carbon-based materials derived from solid waste precursors. The surface is composed of aggregated, irregularly shaped carbonaceous fragments, providing a large number of potential active sites for the adsorption of organic matter and the activation of peroxydisulfate.

After multiple cycles of sludge treatment (Figure 11b,b’), the catalyst retains its overall particle morphology without obvious fragmentation or structural collapse. However, noticeable changes in surface texture are observed: the surface appears more compact and covered with a layer of fine, flaky deposits. These deposits are attributed to the adsorption of sludge organic matter, the formation of inorganic precipitates (e.g., iron phosphate), and the accumulation of reaction by-products on the catalyst surface during the oxidation process [50].

The observed surface fouling may lead to a slight reduction in the accessibility of active sites, which is consistent with the slight decrease in sludge removal rate (from 43.5% to 38%). Importantly, the fundamental particle structure remains intact, indicating that the catalyst has excellent structural stability under the harsh reaction conditions (acidic environment, strong oxidation and mechanical agitation). This stability is crucial for the practical application of the process, as it ensures that the catalyst can be reused multiple times, thereby reducing the overall cost of sludge treatment. The minor surface fouling can be effectively mitigated through simple regeneration methods, such as water washing, which can remove the adsorbed organic and inorganic deposits and restore the catalyst’s activity.

The chemical states and elemental composition of the APPs-derived carbon-based catalyst were analyzed by X-ray photoelectron spectroscopy (XPS). The high-resolution spectra of C 1s, Fe 2p and Cu 2p are shown in Figure 12a–c, respectively.

The C 1s spectrum (Figure 12a) can be deconvoluted into three distinct peaks at 284.6 eV, 286.2 eV and 288.8 eV, corresponding to C–C/C=C, C–O and O–C=O (carbonate) functional groups, respectively [53]. The dominant C–C/C=C peak indicates that the carbon matrix of the catalyst is primarily composed of graphitic carbon, which provides good electrical conductivity and structural stability. The C–O and O–C=O peaks suggest the presence of oxygen-containing functional groups on the catalyst surface [54]. These groups can act as adsorption sites for organic pollutants and also contribute to the electron transfer process, enhancing the catalytic performance of the catalyst.

The Fe 2p spectrum (Figure 12b) exhibits two main peaks at 710.6 eV (Fe 2p_3/2_) and 723.9 eV (Fe 2p_1/2_), with a spin-orbit splitting of ~13 eV. The Fe 2p_3/2_ peak can be deconvoluted into two sub-peaks at 710.8 eV and 713.2 eV, corresponding to Fe^2+^ and Fe^3+^, respectively [55]. The presence of both Fe^2+^ and Fe^3+^ indicates that the iron species in the catalyst exist in a mixed valence state. This is beneficial for the Fenton-like reaction, as Fe^2+^ can activate PDS to generate sulfate radicals (SO_4_·^−^), while Fe^3+^ can be reduced back to Fe^2+^ by the carbon matrix or Cu^+^, thus maintaining the catalytic cycle [56]. The satellite peaks observed in the spectrum further confirm the presence of Fe^3+^ [54].

The Cu 2p spectrum (Figure 12c) shows two broad peaks centered at approximately 933eV (Cu 2p_3/2_) and 953 eV (Cu 2p_1/2_), with a spin-orbit splitting of ~19.75 eV, which is characteristic of copper species [54]. No distinct peak corresponding to metallic Cu^0^ (at ~931.8 eV) was observed. The Cu 2p_3/2_ peak can be deconvoluted into two sub-peaks at 932.5 eV and 933.7 eV, corresponding to Cu^+^ and Cu^2+^, respectively [54]. The coexistence of Cu^+^ and Cu^2+^ is further confirmed by the presence of a satellite peak at ~944 eV, which is a fingerprint of Cu^2+^ [57]. This mixed valence state of Cu might play a crucial role in activating peroxydisulfate and promoting the Fe^2+^/Fe^3+^ redox cycle [58].

The relative content of each chemical state was calculated based on the peak area of the deconvoluted XPS spectra (Table 7). For the C 1s spectrum, the relative contents of C–C/C=C, C–O and O–C=O functional groups were determined to be 68.5%, 18.2% and 13.3%, respectively. The high proportion of C–C/C=C bonds further confirm the dominant graphitic carbon structure of the catalyst, while the non-negligible content of oxygen-containing functional groups (C–O and O–C=O) provides sufficient active sites for adsorption and electron transfer [54]. For the Fe 2p spectrum, the relative contents of Fe^2+^ and Fe^3+^ were calculated as 38.7% and 61.3%, respectively. The higher proportion of Fe^3+^ indicates that Fe species in the catalyst are mainly present in the oxidized state, which can be readily reduced to Fe^2+^ by the carbon matrix or Cu^+^ to sustain the catalytic cycle. In the Cu 2p spectrum, the relative contents of Cu^+^ and Cu^2+^ were 42.5% and 57.5%, respectively. The coexistence of Cu^+^ and Cu^2+^ in a moderate ratio ensures the efficient electron shuttle function of Cu species, which is critical for promoting the Fe^2+^/Fe^3+^ redox cycle and enhancing PDS activation.

### 3.6. Catalyst Stability in Pilot-Scale Operation

**Figure 13:**
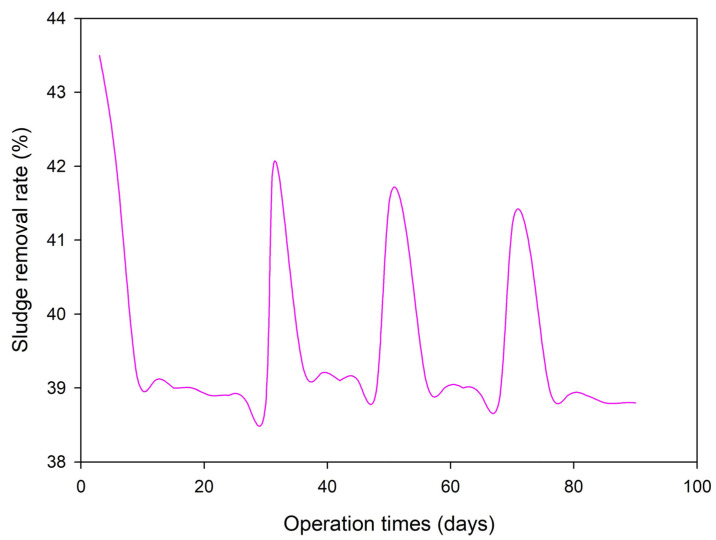
The result of pilot-scale catalyst stability test (90-day operation, with periodic washing).

The stability of the APPs-derived carbon-based catalyst was evaluated through a 90-day continuous pilot-scale operation, with sludge reduction performance tested every 3 days. The results are shown in Figure 13.

As depicted, the sludge removal rate exhibited a characteristic pattern of gradual decline, periodic recovery and re-stabilization throughout the operation. Initially, the sludge removal rate reached 43.5% on day 3, and then gradually decreased to a stable minimum of approximately 38.2% by day 10. This stable low level was maintained until day 30. This initial decline can be attributed to the progressive adsorption of sludge particles and organic matter onto the catalyst surface, which partially blocks the active sites and reduces the accessibility of reactants to the catalytic centers [50].

To mitigate this reversible deactivation, the catalyst was subjected to simple washing with ultrapure water at day 30. Immediately after washing, the sludge removal rate was restored to 41.8%, and after 3 days of re-operation, it remained at 41.3%, a significant recovery of approximately 3 percentage points from the pre-wash stable level. This confirms that the primary cause of deactivation is surface fouling rather than irreversible structural damage. Following this recovery, the removal rate again declined over the subsequent 3 days and re-stabilized at approximately 39% until day 50. A second washing at day 50 resulted in a similar recovery, with the removal rate increasing to 41.5%, followed by a gradual decline back to the stable baseline. A third washing at day 70 produced an identical trend: an initial increase to 41.2%, followed by a 3-day decline and re-stabilization. By the end of the 90-day operation, the sludge removal rate had stabilized at 38.2%, remaining above 38% throughout the entire period, which demonstrates excellent long-term stability.

The excellent recyclability and stability of the catalyst are crucial for the practical application of this process. The periodic washing strategy effectively reverses the surface fouling, reducing the frequency of catalyst replacement and significantly improving the economic efficiency of sludge treatment in long-term operation. This confirms that the APPs-derived catalyst is a robust and viable candidate for industrial-scale sludge reduction applications.

### 3.7. Reaction Mechanism Analysis

#### 3.7.1. Free Radical Pathway in Sludge Degradation

**Figure 14:**
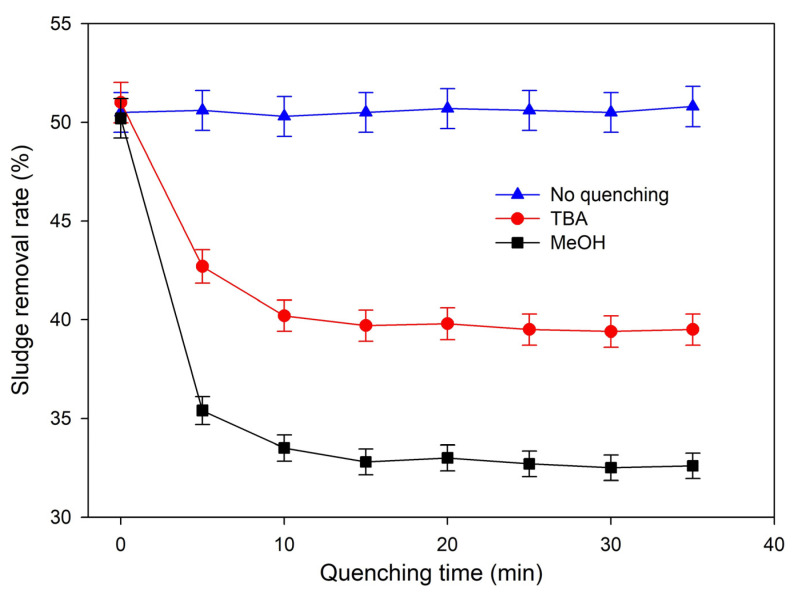
Effects of radical scavengers (methanol and tert-butanol) on sludge removal rate in the ultrasound-Fe^2+^/PDS system (*n* = 3, data are presented as mean ± standard deviation).

Free radical quenching experiments were conducted at the lab-scale to elucidate the roles of sulfate radicals (SO_4_·^−^) and hydroxyl radicals (·OH) in sludge degradation, as well as the auxiliary effect of intrinsic Cu and Fe metals in the APPs-derived catalyst on radical generation. Tert-butanol (TBA) was employed as the specific quencher for ·OH (reaction rate constant with ·OH: 3.8 × 10^8^ L·mol^−1^·s^−1^; with SO_4_·^−^: 4.0 × 10^5^ L·mol^−1^·s^−1^), while methanol (MeOH) served as the dual quencher for both ·OH and SO_4_·^−^ (reaction rate constants with both radicals: ~1.0 × 10^9^ L·mol^−1^·s^−1^) [59]. Under optimal process conditions, quenchers were added at a fixed molar ratio to Na_2_S_2_O_8_, and the sludge removal rate was monitored dynamically over 35 min (Figure 14).

As illustrated in Figure 14, the sludge removal rate stabilized at approximately 51.0% throughout the 35 min reaction in the absence of quenchers, confirming the stable oxidative performance of the synergistic system. In the TBA-added group, the sludge removal rate exhibited a rapid decline in the initial 10 min and then stabilized at 39.5% after 25 min representing a total reduction of 11.5 percentage points relative to the no-quenching group. This significant decline verifies that ·OH participates in sludge degradation and contributes to the oxidation of organic components in sludge flocs and EPS [56].

In contrast, the MeOH-added group showed a more drastic decrease in sludge removal rate: the rate plummeted in the first 5 min and stabilized at 32.5% after 25 min, with a total reduction of 18.5 percentage points compared to the no-quenching group. Since MeOH quenches both SO_4_·^−^ and ·OH, the additional reduction (7% points) beyond the TBA group directly reflects the dominant contribution of SO_4_·^−^. Based on the relative changes in sludge removal rate, SO_4_·^−^ was calculated to contribute approximately 61% of the sludge degradation effect, while ·OH accounted for about 39%, demonstrating that SO_4_·^−^ is the primary reactive radical driving sludge reduction in this system. The primary radical generation reactions in the Fe^2+^/PDS system are summarized as follows [58]:

Fe^2+^ + S_2_O_8_^2−^ → Fe^3+^ + SO_4_·^−^ + SO_4_^2−^ (1)

SO_4_·^−^ + H_2_O → OH + H^+^ + SO_4_^2−^ (2)

SO_4_·^−^ + OH^−^ → ·OH + SO_4_^2−^ (3)

Fe^2+^ acts as the primary activator of PDS, reacting with S_2_O_8_^2−^ to generate SO_4_·^−^ (Equation (1)). A portion of the generated SO_4_·^−^ further converts to ·OH via hydrolysis or reaction with hydroxyl ions (Equations (2) and (3)). Both SO_4_·^−^ (E^0^ = 2.5–3.1 V) and ·OH (E^0^ = 2.8 V) possess strong oxidizing capacity, capable of cleaving key chemical bonds (C-H, C-C, C-O) in EPS and organic pollutant molecules. This cleavage degrades macromolecular organics into small-molecule intermediates and partially mineralizes organics to CO_2_ and H_2_O [48], ultimately achieving efficient sludge reduction.

The intrinsic Cu and Fe metals from waste APPs in the catalyst play a critical auxiliary role in radical generation through two key mechanisms. First, the transition metal species directly activate PDS to produce additional SO_4_·^−^, as described by Equations (4) and (5) [56,58]:

Cu^2+^ + S_2_O_8_^2−^ → Cu^+^ + 2SO_4_^2−^ (4)

Fe^3+^ + S_2_O_8_^2−^ → Fe^2+^ + SO_4_·^−^ + SO_4_^2−^ (5)

Second, the Cu^2+^/Cu^+^ redox pair in the catalyst accelerates the Fe^3+^/Fe^2+^ cycle (Equation (6)), maintaining a high effective concentration of Fe^2+^ in the system, promoting continuous PDS activation, and increasing the total radical yield [60]:

Cu^+^ + Fe^3+^ → Cu^2+^ + Fe^2+^ (6)

This auxiliary activation effect of the APPs-derived catalyst significantly elevates the PDS activation efficiency and radical generation capacity of the system. It is the core reason why the synergistic system achieves a far superior sludge reduction effect compared to the single Fe^2+^/PDS system, fully validating the high catalytic value of this waste APPs-derived resource product.

#### 3.7.2. Non-Free Radical Pathway in Sludge Degradation

In addition to the free radical pathway, the APPs-derived carbon-based catalyst might also activate PDS through a non-free radical mechanism, which is a critical contributor to the enhanced sludge degradation efficiency. To verify the existence and quantify the contribution of the non-free radical pathway, a series of control experiments were conducted in the presence of excess TBA (TBA:Na_2_S_2_O_8_ = 10:1). TBA was used to completely quench all generated ·OH radicals, thereby isolating the contribution of non-free radical and SO_4_·^−^-mediated oxidation. The test results were shown in Table 8.

The significantly higher sludge removal rate observed in the synergistic system, compared to the single Fe^2+^/PDS system under the same quenching conditions, confirms the existence of a distinct non-free radical pathway. This pathway contributes approximately 10.8% to the total sludge degradation effect. Importantly, the non-free radical pathway offers unique advantages, such as strong anti-interference ability and high selectivity for organic pollutant degradation. It effectively mitigates the drawback of free radicals being easily consumed by side reactions in the complex sludge matrix [30].

This pathway is primarily enabled by the catalyst’s unique physicochemical properties [61]: a porous carbon framework, abundant surface defect sites and a high density of oxygen-containing functional groups (C-O: 18.2%, O–C=O: 13.3%), as confirmed by XPS analysis. These features collectively create a favorable microenvironment for PDS activation without necessarily generating free radicals in the bulk solution. Briefly, PDS is enriched on the catalyst surface, and electrons from the carbon matrix and Cu/Fe redox sites activate PDS into surface-bound reactive complexes, which oxidize sludge organics in situ.

The intrinsic Cu and Fe metals in the catalyst play a crucial auxiliary role in this non-free radical pathway. Cu^2+^ acts as an efficient electron shuttle, accepting electrons from the carbon surface and transferring them to PDS, thereby accelerating the electron transfer process. Meanwhile, Fe^3+^ forms surface complexes with the oxygen-containing functional groups, which modifies the electronic structure of the catalyst surface and enhances its adsorption capacity for PDS, indirectly promoting the non-free radical activation [54].

#### 3.7.3. Overall Synergistic Mechanism

In summary, the outstanding sludge reduction performance in this pilot-scale system can be attributed to the synergistic effect of free radical and non-free radical oxidation pathways, both of which are significantly promoted by the APPs-derived carbon-based catalyst.

The free radical pathway, dominated by sulfate radicals (SO_4_·^−^) and supplemented by hydroxyl radicals (·OH), acts as the primary driving force for the oxidative degradation of sludge organic matter. The intrinsic Cu and Fe species in the catalyst not only directly activate peroxydisulfate but also effectively accelerate the Fe^2+^/Fe^3+^ redox cycle, ensuring continuous and efficient generation of reactive radicals, thereby achieving strong oxidation and decomposition of EPS and organic pollutants.

Meanwhile, the non-free radical pathway, based on surface adsorption, direct electron transfer, and surface catalytic reactions, serves as a stable and high-selectivity supplementary route. It effectively compensates for the interference susceptibility of free radicals in complex sludge matrices and improves the overall anti-interference ability and operational stability of the system, contributing approximately 10.8% to the total sludge degradation efficiency.

The combination of these two pathways forms a highly efficient, stable and comprehensive oxidation system, which is the fundamental reason for the superior sludge reduction effect, long-term operational stability and environmental safety of the proposed APPs-derived catalyst/ultrasound-Fe^2+^/PDS process. This synergistic mechanism not only realizes the high-value resource utilization of waste antifouling paint particles but also provides a novel, green and engineering-feasible technical route for sustainable municipal sludge treatment.

## 4. Conclusions

This study successfully constructed a novel activated sludge reduction process coupling APPs-derived carbon-based catalysts with ultrasound-Fe^2+^/S_2_O_8_^2−^ advanced oxidation in pilot-scale, realizing high-value resource utilization of waste APPs from ship derusting wastewater and deep reduction in activated sludge simultaneously.

Through parameter optimization, multi-dimensional characterization and mechanism analysis, it was confirmed that the integrated system achieves efficient sludge reduction via the synergistic effect of ultrasonic cavitation, Fe^2+^/PDS oxidation and APPs-derived catalyst catalysis. The catalyst, prepared using waste APPs as the sole raw material, retains intrinsic Cu/Fe mixed valence metals as core active components, enhancing persulfate activation through free radical (dominated by SO_4_·^−^) and non-free radical dual pathways, while promoting Fe^2+^/Fe^3+^ cycle regeneration. The catalyst exhibits excellent operational stability during long-term pilot operation, ensuring high reliable engineering applicability.

This “treating waste with waste” process integrates APPs resource utilization and sludge deep reduction, with advantages of high efficiency, low cost and environmental friendliness, providing a new low-carbon technical route for municipal sludge disposal and solving APPs pollution from ship derusting.

Despite the promising results, this study has certain limitations: (1) the pilot-scale operation was conducted under fixed operating conditions, and the adaptability of the process to fluctuating sludge properties (e.g., organic content, moisture content) in actual engineering scenarios was not fully verified; (2) the catalyst preparation process still has room for optimization, as its catalytic activity and stability have not yet reached the level of commercial catalysts; (3) the economic benefit analysis was preliminary, and the long-term operation cost, including catalyst regeneration, energy consumption and sludge post-treatment, needs further quantification.

Future research will focus on three aspects: (1) optimize the APPs-derived catalyst preparation process (e.g., adjust carbonization parameters, introduce modification methods) to further improve its catalytic activity; (2) conduct engineering scale-up experiments, explore the process adaptability to different sludge types and fluctuating operating conditions; (3) carry out in-depth economic and environmental impact assessments, optimize the process flow to reduce operation costs, and provide a comprehensive theoretical and technical basis for the industrial promotion of this process.

## Figures and Tables

**Table 1:** Basic characteristics of raw sludge.

Parameter	Value
Water content (%)	98.2
pH	6.8
Viscosity (m·Pa·s)	10.3
Density (g·mL^−1^)	1.03
Solid density (g·mL^−1^)	1.32
VSS/SS (%)	72.0
SCOD/TCOD (%)	90.1
BOD_5_/COD (%)	53.7
Particle size (*d*_0.5_, μm)	45.73
Zeta potential (mV)	−17.95

**Table 2:** Relationship between sludge flow rate and ultrasonic residence time.

Sludge flow rate (m^3^·h^−1^)	1.0	1.5	2.0	2.5	3.0
Residence time (s)	54	36	27	21.6	18

Note: The effective reaction volume of the six-stage ultrasonic reactor is 15 L.

**Table 3:** Effect of aeration rate on sludge reduction performance ^a^.

NO.	Aeration Rate (m^3^·h^−1^)	Sludge Removal Rate (%)	SCOD/TCOD (%)	VSS/SS (%)
1 ^b^	0 (no catalyst)	28.2	57.5	45.2
2	0 (with catalyst)	32.5	44.1	36.4
3 ^c^	3.5	-	-	-
4	4.5	41.7	23.2	17.7
5	5.5	43.4	19.8	16.2
6	6.5	43.2	20.3	15.9
7 ^d^	7.5	-	-	-

Notes: ^a^. FeSO_4_ = 40 mol·m^−3^, Na_2_S_2_O_8_ = 45 mol·m^−3^, catalyst dosage = 650 kg, sludge flow rate = 1.0 m^3^·h^−1^, ultrasonic parameters were optimal; ^b^. No catalyst was loaded in the reactor; other conditions were the same as a; ^c^. The air blower operated unstable with surge at aeration rate ≤ 3.5 m^3^·h^−1^; ^d^. A small amount of catalyst particles was observed in the effluent at aeration rate ≥ 7.5 m^3^·h^−1^, causing catalyst loss (loss rate > 5%).

**Table 4:** Relationship between catalyst dosage, bulk volume and volume ratio.

Catalyst Dosage (kg)	Catalyst Bulk Volume (m^3^)	Bulk Volume/Effective Volume (%)
550	0.76	28.0
600	0.83	30.6
650	0.90	33.2
700	0.97	35.7
750	1.04	38.4

Note: The bulk density of the APPs-derived carbon-based catalyst was 0.72 kg m^−3^, and the material density was 1.05 kg m^−3^, all derived from waste APPs, realizing the complete resource retention of metal elements in waste.

**Table 5:** Orthogonal test results (L_9_(3^3^)).

Test No.	FeSO_4_ Concentration (mol m^−3^)	Catalyst Dosage (kg)	Aeration Rate (m^3^ h^−1^)	Sludge Removal Rate (%)
1	40	600	4.5	38.5
2	40	650	5.5	42.8
3	40	700	6.5	40.3
4	60	600	5.5	42.1
5	60	650	6.5	43.5
6	60	700	4.5	41.2
7	80	600	6.5	39.8
8	80	650	4.5	38.9
9	80	700	5.5	40.5

**Table 6:** Sludge particle size and Zeta potential at different treatment stages.

Treatment Stage	Particle Size d_0.5_ (μm)	Zeta Potential (mV)
Raw sludge	45.73	−17.95
After ultrasonic pretreatment	32.16	−15.28
After integral synergistic process	18.79	−8.63

**Table 7:** XPS peak fitting results of the APPs-derived carbon-based catalyst.

Spectral Orbital	Peak Assignment	Peak Area	Relative Content (%)
C 1s	C–C/C=C	6850	68.5
	C–O	1820	18.2
	O–C=O	1330	13.3
Fe 2p	Fe^2+^ (Fe 2p_3/2_, Fe 2p_1/2_)	3870	38.7
	Fe^3+^ (Fe 2p_3/2_, Fe 2p_1/2_)	6130	61.3
Cu 2p	Cu^+^ (Cu 2p_3/2_, Cu 2p_1/2_)	4250	42.5
	Cu^2+^ (Cu 2p_3/2_, Cu 2p_1/2_)	5750	57.5

Note: The relative content of each chemical state was calculated based on the integrated peak area of the deconvoluted XPS spectra.

**Table 8:** Sludge removal rates of different systems in the presence of excess TBA (to quench ·OH).

System	Sludge Removal Rate (%)
APPs-catalyst + Fe^2+^/PDS + excess TBA	25.2
Single Fe^2+^/PDS system + excess TBA	20.5
Contribution of non-free radical pathway	10.8%

Note: Tert-butanol (TBA) was used as a specific scavenger to quench hydroxyl radicals (·OH). The contribution of the non-free radical pathway was calculated as follows: Net non-free radical contribution = (Sludge removal rate of APPs-catalyst + Fe^2+^/PDS + excess TBA) − (Sludge removal rate of single Fe^2+^/PDS + excess TBA) = 25.2% − 20.5% = 4.7%; Contribution ratio = (Net non-free radical contribution/Total sludge removal rate of APPs-catalyst + Fe^2+^/PDS system under optimal conditions) × 100% = (4.7%/43.5%) × 100% = 10.8%.

## Data Availability

The original contributions presented in this study are included in the article. Further inquiries can be directed to the corresponding author.

## Supplementary Materials

The following supporting information can be downloaded at: https://www.mdpi.com/article/10.3390/toxics14040292/s1. Text S1: Collection of Ship Derusting Wastewater and Extraction of APPs. Text S2: Preparation of APPs-Derived Carbon-Based Catalyst. Table S1: Analytical methods and instruments for sludge physicochemical indices.

## Abbreviations

The following abbreviations are used in this manuscript:

PDS	Peroxydisulfate
APPs	Antifouling Paint Particles
SEM	Scanning Electron Microscopy
EDS	Energy Dispersive Spectroscopy
XPS	X-ray Photoelectron Spectroscopy
TN	Total Nitrogen
TP	Total Phosphorus
EPS	Extracellular Polymeric Substances
IMO	International Maritime Organization
TBA	Tert-butanol
BET	Brunauer Emmett Teller
SS	Suspended Solids
MLSS	Mixed Liquor Suspended Solids
VSS	Volatile Suspended Solids
COD	Chemical Oxygen Demand
SCOD	Soluble Chemical Oxygen Demand
TCOD	Total Chemical Oxygen Demand
NH_3_-N	Ammonia Nitrogen

## References

[1] Yuan H., Cheng X., Chen S., Zhu N., Zhou Z. (2011). New sludge pretreatment method to improve dewaterability of waste activated sludge. Bioresour. Technol..

[2] Citeau M., Larue O., Vorobiev E. (2011). Influence of salt, pH and polyelectrolyte on the pressure electro-dewatering of sewage sludge. Water Res..

[3] Feng Y., Yu T., Chen D., Xu G., Wan L., Zhang Q., Hu Y. (2017). Effect of Hydrothermal Treatment on the Steam Gasification Behavior of Sewage Sludge: Reactivity and Nitrogen Emission. Energy Fuels.

[4] Zhou S., Wu M., Chen Z., Yang Y., Zhi D. (2025). Current treatment techniques for landfill leachate: Mechanisms, influencing factors, performance, and prospects. Environ. Monit. Assess..

[5] Pili S., Bhunia P., Yan S., Leblanc R.J., Tyagi R.D., Surampalli R.Y. (2011). Ultrasonic pretreatment of sludge: A review. Ultrason. Sonochem..

[6] Xiao J., Guo S., Wang D., An Q. (2024). Fenton-Like Reaction: Recent Advances and New Trends. Chemistry.

[7] Zheng T.H., Zhang Z.Z., Liu Y., Zou L.H. (2025). Recent Progress in Catalytically Driven Advanced Oxidation Processes for Wastewater Treatment. Catalysts.

[8] Xu W., Li S., Huang D., Cheng M., Wang G., Du L., Li R. (2025). Dual nonradical pathways in enhanced PDS activation by Fe@BC-S: Fe_7_S_8_ mediated the relationship between ^1^O_2_ and electron transfer. Chem. Eng. J..

[9] Ramirez-de-Arellano J.M., Canales M., Magaña L.F. (2021). Carbon Nanostructures Doped with Transition Metals for Pollutant Gas Adsorption Systems. Molecules.

[10] Parts H., Stamatakis M. (2024). Transition Metal Carbides as Supports for Catalytic Metal Particles: Recent Progress and Opportunities. J. Phys. Chem. Lett..

[11] Sørensen P.A., Kiil S., Dam-Johansen K., Weinell C.E. (2009). Anticorrosive coatings: A review. J. Coat. Technol. Res..

[12] Oni B.A., Tomomewo O.S., Evro S., Misiani A.N., Sanni S.E. (2025). A review of anticorrosive, superhydrophobic and self-healing properties of coating-composites as corrosion barriers on magnesium alloys: Recent advances, challenges and future directions. J. Magnes. Alloys.

[13] Muller-Karanassos C., Turner A., Arundel W., Vance T., Lindeque P.K., Cole M. (2019). Antifouling paint particles in intertidal estuarine sediments from southwest England and their ingestion by the harbour ragworm, *Hediste diversicolor*. Environ. Pollut..

[14] Soroldoni S., Castro I.B., Abreu F., Duarte F.A., Choueri R.B., Möller O.O., Fillmann G., Pinho G.L.L. (2018). Antifouling paint particles: Sources, occurrence, composition and dynamics. Water Res..

[15] Banerjee I., Pangule R.C., Kane R.S. (2011). Antifouling coatings: Recent developments in the design of surfaces that prevent fouling by proteins, bacteria, and marine organisms. Adv. Mater..

[16] Suresh J., Subbaiyan R., Ganesan A., Ramasubramanian B. (2024). Effective antifouling action of seaweed metabolite waste against marine foulers. Sustain. Chem..

[17] Li X., Zhu X., Zhang Y., Cao P., Wang R., He Y. (2022). Cationic copolymer Sweetsop-shape nanospheres conjugating SalPhen-Zinc complex for excellent antimicrobial. Eur. Polym. J..

[18] Hail S.B., Nikles D.E. (2000). Synthesis and characterization of copoly(acrylate)s with silane functional groups in the side chain. Polym. Prepr..

[19] Soroldoni S., Abreu F., Castro í.B., Duarte F.A., Pinho G.L.L. (2017). Are antifouling paint particles a continuous source of toxic chemicals to the marine environment?. J. Hazard. Mater..

[20] Turner A., Pollock H., Brown M.T. (2009). Accumulation of Cu and Zn from antifouling paint particles by the marine macroalga, *Ulva lactuca*. Environ. Pollut..

[21] Howell D., Behrends B. (2006). A methodology for evaluating biocide release rate, surface roughness and leach layer formation in a TBT-free, self-polishing antifouling coating. Biofouling.

[22] Chen Y.J., Chen Z.S., Zhao W.T., Huang L.Y. (2024). Optimisation strategy to enhance the performance and efficiency of self-rotary water-jet derusting sprayers. Ocean Eng..

[23] Chen Z.S., Zhao W.T., Li J.L., Chen Y.J., Huang L.Y. (2025). An advanced framework for the layout optimization of multi-nozzle rotary derusting sprayer using CFD-IBES methods. Appl. Ocean Res..

[24] Kim N.S., Shim W.J., Yim U.H., Ha S.Y., Park P.S. (2008). Assessment of tributyltin contamination in a shipyard area using a mussel transplantation approach. Mar. Pollut. Bull..

[25] Jaini M., Namboothri N. (2023). Boat paint and epoxy fragments-Leading contributors of microplastic pollution in surface waters of a protected Andaman bay. Chemosphere.

[26] Boucher J., Friot D. (2017). Primary Microplastics in the Oceans: A Global Evaluation of Sources.

[27] Costa M.F., Barletta M. (2015). Microplastics in coastal and marine environments of the western tropical and sub-tropical Atlantic Ocean. Environ. Sci. Process. Impacts.

[28] Li S.Q., Wang H., Feng X.Y., Zeng Y., Shen Y., Gu Q. (2025). Microplastics in Chinese coastal waters: A mini-review of occurrence characteristics, sources and driving mechanisms. Waste Manag. Res..

[29] Gao L., Guo Y., Zhan J., Yu G., Wang Y. (2022). Assessment of the validity of the quenching method for evaluating the role of reactive species in pollutant abatement during the persulfate-based process. Water Res..

[30] Zhou Y., Guo W., Li Y., Gao M., Li X., Liu W., Chen Z., Zhang X., Zhou Y., Xing M. (2025). Insights into free radical and non-radical routes regulation for water cleanup. Nat. Commun..

[31] Liang H., Shi X., Li Y. (2024). Technologies in Marine Antifouling and Anti-Corrosion Coatings: A Comprehensive Review. Coatings.

[32] Qiu Q., Gu Y., Ren Y., Ding H., Hu C., Wu D., Mou J., Wu Z., Dai D. (2024). Research progress on eco-friendly natural antifouling agents and their antifouling mechanisms. Chem. Eng. J..

[33] Zhu P., Feng D., Yasir M., Song W., Hafeez M.A., Zhang C., Liu L. (2023). Enhanced antifouling capability of PDMS/Cu_2_O-anchored Fe-based amorphous coatings. Surf. Coat. Technol..

[34] El Jery A., Kosarirad H., Taheri N., Bagheri M., Aldrdery M., Elkhaleefa A., Wang C., Sammen S.S. (2023). An Application of Ultrasonic Waves in the Pretreatment of Biological Sludge in Urban Sewage and Proposing an Artificial Neural Network Predictive Model of Concentration. Sustainability.

[35] Izadifar Z., Babyn P., Chapman D. (2019). Ultrasound Cavitation/Microbubble Detection and Medical Applications. J. Med. Biol. Eng..

[36] Meng Z., Zhou Z., Zheng D., Liu L., Dong J., Yang Y., Li X., Zhang T. (2018). Optimizing dewaterability of drinking water treatment sludge by ultrasound treatment: Correlations to sludge physicochemical properties. Ultrason. Sonochem..

[37] Margulis I.M., Polovinkin V.N., Yashin A.I. (2024). Modern Approaches to the Description of the Dynamics of Cavitation Bubbles and Cavitation Clouds. Tech. Phys. Lett..

[38] Zhang H., Du S., Wang Y., Xue F. (2024). Prevention of Crystal Agglomeration: Mechanisms, Factors, and Impact of Additives. Crystals.

[39] Bian C., Ge D., Wang G., Dong Y., Li W., Zhu N., Yuan H. (2021). Enhancement of waste activated sludge dewaterability by ultrasound-activated persulfate oxidation: Operation condition, sludge properties, and mechanisms. Chemosphere.

[40] Xu L., Zhang Z., Graham N.D.J., Yu W. (2024). Exploring the influence of aquatic phosphate on Fe floc dynamics in water treatment. Water Res..

[41] Xu H., Ma Y., Chen J., Zhang W., Ynag J. (2022). Electrocatalytic reduction of nitrate—A step towards a sustainable nitrogen cycle. Chem. Soc. Rev..

[42] Xia J., Ji J., Hu Z., Rao T., Liu A., Ma J., Sun Y. (2022). Application of Advanced Oxidation Technology in Sludge Conditioning and Dewatering: A Critical Review. Int. J. Environ. Res. Public Health.

[43] Mdolo P., Pocock J., Velkushanova K. (2024). Optimization of the Solubilization of Faecal Sludge through Microwave Treatment. Water.

[44] Yu H.Q. (2020). Molecular Insights into Extracellular Polymeric Substances in Activated Sludge. Environ. Sci. Technol..

[45] Long A., Yang L., Zhang H. (2014). Degradation of Toluene by a Selective Ferrous Ion Activated Persulfate Oxidation Process. Ind. Eng. Chem. Res..

[46] Zhang T., Chen Y., Wang Y., Le Roux J., Yang Y., Croué J.P. (2014). Efficient peroxydisulfate activation process not relying on sulfate radical generation for water pollutant degradation. Environ. Sci. Technol..

[47] Lv Z., You H., Xu M., Leng H., Li W., Zhao Z., Li Y., Zhu J., Zhang G. (2024). Synergetic sludge conditioning by US enhanced Fe^2+^ activated sodium persulfate: Physicochemical properties and mechanisms. Chemosphere.

[48] Hou J., Hong C., Ling W., Hu J., Feng W., Xing Y., Wang Y., Zhao C., Feng L. (2024). Research progress in improving sludge dewaterability: Sludge characteristics, chemical conditioning and influencing factors. J. Environ. Manag..

[49] Golbabaei Kootenaei F., Mehrdadi N., Nabi Bidhendi G., Amini Rad H., Hasanlou H., Mahmoudnia A. (2022). Improvement of Sludge Dewatering by Ultrasonic Pretreatment. Int. J. Environ. Res..

[50] Zhang H., Tu C., He C. (2025). Study on Sustainable Sludge Utilization via the Combination of Electroosmotic Vacuum Preloading and Polyacrylamide Flocculation. Sustainability.

[51] Huang J., Fu Q., Shao X., Li Y. (2025). Ultrasonic strategies for mitigating microbial adhesion and biofilm formation on medical surfaces: A mini review. Front. Microbiol..

[52] Yang Y., Yang X., Chen Y., Li X., Yang Q., Li Y., Ma P., Zhang H., Xu S. (2024). Response surface optimization of sludge dewatering process: Synergistic enhancement by ultrasonic, chitosan and sludge-based biochar. Water. Sci. Technol..

[53] Li W., Xie B., Li Z., Zhang G., Lv Z., Li Q., Zhao S., Tong M., Gao Y., Bai L. (2026). Ultrasonic enhanced Fe-C/PDS system for sludge dewatering and carbon source release: Synergy of oxidation and cracking processes. Bioresour. Technol..

[54] Zhao Z., Cai X., Fan S., Zhang Y., Huang Z., Hu H., Liang J., Qin Y. (2021). Construction of a stable Cu-Fe@C composite catalyst with enhanced performance and recyclability for visible-light-driven photo-Fenton reaction. J. Alloys Compd..

[55] Greczynski G., Hultman L. (2021). The same chemical state of carbon gives rise to two peaks in X-ray photoelectron spectroscopy. Sci. Rep..

[56] Ling C., Huang R., Mao W., Wu Z., Wei C., Li A., Zhou J. (2025). Activation of H_2_O_2_/PDS/PMS by Iron-Based Biochar Derived from Fenton Sludge for Oxidative Removal of 2,4-DCP and As (III). Water.

[57] Conradie J., Erasmus E. (2022). XPS photoelectron lines, satellite structures and Wagner plot of Cu (II) β-diketonato complexes explained in terms of its electronic environment. J. Electron Spectrosc. Relat. Phenom..

[58] Dong L., Lan X., Ying D., E Q., Yan J., Tian H., Zhao H., Huang Y., Fang Y. (2025). Efficient activation of peroxydisulfate by Cu-biochar hybrid materials: Source and transfer direction of interface electrons. Sep. Purif. Technol..

[59] Bai M., Li M., Ding X., Wang Y. (2021). Quenching effect of different quenchers on SO^4−^ and ·OH based advanced oxidation processes. Ind. Water Treat..

[60] Zhong C., Jiao Q., Wen J., Liu R., Zhao X., Geng J., Liu J., Feng Y. (2025). e-Cu bimetallic synergy boosts Fe^2+^ regeneration and electron transfer for ^1^O_2_-dominated peroxymonosulfate activation. J. Water Process. Eng..

[61] Luo X., Yuan P., Luo J., Xiao H., Li J., Zheng H., Du B., Li D., Chen Y. (2023). The Enhancing Effect of Stable Oxygen Functional Groups on Porous-Carbon-Supported Pt Catalysts for Alkaline Hydrogen Evolution. Nanomaterials.

