# Supplementary material for: Application of Carbon-Based Catalysts Derived from Ship Antifouling Paint Particles in Ultrasound-Fe2+/Peroxydisulfate Advanced Oxidation Process for Activated Sludge Reduction: A Pilot-Scale Study
Source: Toxics. 2026 Mar 28;14(4):292. doi: 10.3390/toxics14040292 (PMC13120440; doi:10.3390/toxics14040292)
Supplement: Supplementary file 1 [file toxics-14-00292-s001.zip › toxics-4220646-supplementary.pdf]

Application of Carbon-Based Catalysts Derived from Ship Antifouling Paint Particles in Ultrasound-Fe^{2+}/Peroxydisulfate Advanced Oxidation Process for Activated Sludge Reduction: A Pilot-Scale Study

Supplementary information

Text S1. Collection of Ship Derusting Wastewater and Extraction of APPs

1. Sampling of Derusting Wastewater

Derusting wastewater samples were collected during the ultra-high-pressure water jet derusting process of an 82,000 deadweight tonnage (dwt) bulk carrier in a dry dock of a ship repair yard in Nantong, China. The bulk carrier had an overall length of 225 m, molded width of 36 m, molded depth of 19.2 m, full-load draft of 13.8 m, and a total hull surface area of approximately 15,600 m^2. Eight ultra-high-pressure water jet robots were operated simultaneously for derusting, with each robot having a water output of 50 $L \cdot min^{-1}$ and a hull surface treatment capacity of up to 60 $m^2 \cdot h^{-1}$.

Derusting wastewater, dominated by suspended solid phases of APPs and surface-adhered iron rust, was collected through a closed vacuum recovery system and continuously transported to a dedicated centralized collection tank (effective volume: 5 m^3) via a special main pipeline. The solid content of the derusting wastewater was determined to be 22 ± 1.7 g L^{-1}. To obtain a large amount of representative APPs raw materials for subsequent carbon-based catalyst preparation, a total of 50 m^3 of well-mixed derusting wastewater was collected in batches during the stable derusting stage (excluding the initial short-term pipeline flushing phase and the final rinsing phase).

2. Extraction and Pretreatment of APPs

Given that derusting wastewater is mainly composed of suspended APPs and iron rust with no complex organic impurity interference, APPs were extracted via a simple solid-liquid separation combined with magnetic separation method to achieve large-scale collection of high-purity raw APPs. The specific extraction and pretreatment steps were as follows:

Continuous solid–liquid separation: the 50 m^3 collected derusting wastewater was first subjected to gravity sedimentation in a closed sedimentation tank for 3 h to realize preliminary separation of the solid and liquid phases, and the supernatant with low solid content (solid content < 0.3 gL^{-1}) was discarded. The concentrated slurry containing a high content of APPs and iron rust was then pumped into a plate-and-frame filter press for pressure filtration (filter pressure: 0.6 MPa, filter membrane: polypropylene filter cloth with a pore size of 5 μm) to complete efficient solid–liquid separation, and the filter cake (moisture content: ~65%) was collected.

Magnetic separation of iron rust: the collected filter cake was spread in a clean air-drying tray and naturally air-dried to a constant weight at room temperature (25 ± 2 °C) in a well-ventilated environment to remove free water. The dried solid mixture was subjected to magnetic separation using a high-intensity magnetic separator (magnetic field strength: 1.2 T) to remove iron rust impurities; the non-magnetic fraction (mainly APPs) was collected with a recovery rate of 82.3 ± 3.1%.

Mechanical crushing and grading: the non-magnetic fraction was crushed using a high-speed universal crusher and then subjected to mechanical screening using a standard test sieve (150 μm aperture) to remove a small amount of large-diameter impurities; the undersize fraction was further sieved using a 150-mesh filter to collect the <100 μm particle size fraction as the final APPs raw material for carbon-based catalyst preparation. This particle size range was selected to ensure the uniformity of the catalyst precursor and the formation of a porous structure during subsequent carbonization.

Through the above process, a total of 814.6 kg of APPs raw material which fully met the demand for catalyst preparation and the related characterization tests of APPs.

Text S2. Preparation of APPs-derived carbon-based catalyst

A carbonization-molding method was adopted to prepare columnar carbon-based catalysts, with the core innovation of using APPs as the sole raw material (no additional activated carbon or metal compounds were added), realizing the complete resource conversion

of waste APPs without secondary waste generation. The detailed preparation steps were as follows:

Optimization of molding aid dosage: starch was used as the molding aid, and different addition amounts (3 wt%, 5 wt%, 7 wt%) were compared. The results showed that when the starch addition amount was 5 wt%, the prepared columnar catalyst had the best mechanical strength (compressive strength: 12.5 MPa) and fluidization performance. Therefore, 5 wt% starch was selected as the optimal molding aid dosage.

Molding: 5 wt% starch was added to the dried APPs and mixed uniformly, followed by the addition of an appropriate amount of ultrapure water to form a plastic mud. Starch only played a shaping role and did not participate in the catalytic reaction, ensuring that all active components of the catalyst were derived from APPs.

Extrusion molding: the plastic mud was transferred to a tablet press and pressed into columnar particles with a diameter of 5 mm and a length of 10–15 mm under a pressure of 15 MPa. The columnar shape facilitated the fluidization of the catalyst in the fluidized bed reactor and improved its contact efficiency with sludge.

Carbonization: the columnar APPs particles were placed in a tube furnace and carbonized under a high-purity nitrogen (N_2) atmosphere (flow rate: 100 $mL\cdot min^{-1}$). The carbonization temperature was optimized by comparing the Methylene Blue degradation activity of catalysts + peroxydisulfate (PDS) prepared at 300 °C, 400 °C, 500 °C and 600 °C, and the results showed that the catalyst prepared at 500 °C had the highest PDS activation efficiency. Therefore, the temperature was raised to 500 °C at a heating rate of 5 $°C\cdot min^{-1}$ and held constant for 2 h. After natural cooling to room temperature, broken particles were removed by sieving to obtain the final APPs-derived columnar carbon-based catalyst.

Table S1. Analytical methods and instruments for sludge physicochemical indices.

Parameter	Instrument	Method
Water content (%)	DHG-9070A Oven (Donglu Instrument Equipment, Shanghai, China), FA2004 Analytical balance (Jingtian Electronic Instruments, Shanghai, China)	Gravimetric method (105 °C, 24 h)
pH	S400-K pH meter (METTLER TOLEDO, Greifensee, Switzerland)	Glass electrode method
Viscosity (mPa·s)	VTE-250 viscosimeter (AMETEK BrookField, Middleboro, MA, USA)	Rotational viscometry
Density (g·mL^{-1})	Densitometer D6 (METTLER TOLEDO, Greifensee, Switzerland)	Pycnometer method
Solid density (g·mL^{-1})	Pycnometer	Pycnometer method
Volatile Suspended Solids / Suspended Solids (VSS/SS) (%)	DHG-9070A Oven (Donglu Instrument Equipment, Shanghai, China), SX2-4-10 Muffle furnace (Kailang Instrument and Equipment, Shanghai, China), FA2004 Analytical balance (Jingtian Electronic Instruments, Shanghai, China)	Gravimetric method (550 °C, 2 h)
Soluble Chemical Oxygen Demand / Total Chemical Oxygen Demand (SCOD/TCOD) (%)	KLB-100 Chemical Oxygen Demand (COD) rapid analyzer (Shandong Ketong, Zibo, Shandong, China)	Dichromate oxidation method

Total phosphorus (TP)	UV-2600 UV–Vis spectrophotometer (Tianmei Yituo Laboratory Equipment, Shanghai, China)	Molybdenum antimony resistance colorimetric method
Total nitrogen (TN)	UV-2600 UV–Vis spectrophotometer (Tianmei Yituo Laboratory Equipment, Shanghai, China)	Alkaline potassium persulfate digestion-UV spectrophotometric method
Ammonia nitrogen (NH_3-N)	UV-2600 UV–Vis spectrophotometer (Tianmei Yituo Laboratory Equipment, Shanghai, China)	Nessler's reagent spectrophotometric method
Toluene	GC-2014 Gas chromatograph (Shimadzu, Nakagyo-ku, Kyoto, Japan)	Headspace gas chromatography
Phenolic compounds	UV-2600 UV–Vis spectrophotometer (Tianmei Yituo Laboratory Equipment, Shanghai, China)	4-Aminoantipyrine spectrophotometric method
